# NMR, FT-IR, XRD, SEM, and ANN Complex Characterization of Some Nonwoven Materials Produced by Electrospinning

**DOI:** 10.3390/ma18214893

**Published:** 2025-10-25

**Authors:** Ramona Crainic, Petru Pășcuță, Florin Popa, Radu Fechete

**Affiliations:** 1 Doctoral School, Faculty of Physics, Babeş-Bolyai University, 1 Kogălniceanu, 400084 Cluj-Napoca, Romania; ramona.crainic95@gmail.com; 2 Faculty of Material and Environmental Engineering, Technical University of Cluj-Napoca, 103-105 Muncii, 400641 Cluj-Napoca, Romania; petru.pascuta@phys.utcluj.ro (P.P.); florin.popa@stm.utcluj.ro (F.P.)

**Keywords:** electrospun nanofibers, chitosan, marine fish collagen and fish gelatin biopolymers, PVA and PEG polymers, 1D and 2D ^1^H NMR relaxometry and spectroscopy, FT-IR spectroscopy, SEM, DRX, SEM-ANN prediction of nanofibers’ order degree

## Abstract

Electrospinning is a versatile technique used to manufacture nanofibers by applying an electric field to a polymer solution. This method has gained significant interest in the biomedical, pharmaceutical, and materials engineering fields due to its ability to produce porous structures with a high specific surface area, making it ideal for applications such as wound dressings, controlled drug delivery systems, and tissue engineering. The materials used in electrospinning play a crucial role in determining the final properties of the obtained nonwoven nanofibers. Among the most studied substances are chitosan, collagen, and fish-derived gelatin, which are biopolymers with high biocompatibility. These materials are especially used in the medical and pharmaceutical fields due to their bioactive properties. In combination with synthetic polymers such as polyethylene glycol (PEG) and polyvinyl alcohol (PVA), these biopolymers can form electrospun fibers with improved mechanical characteristics and enhanced structural stability. The characterization of these materials was performed using modern characterization techniques, such as one-dimensional (1D) proton NMR spectroscopy (^1^H), for which the spin–spin relaxation time distributions *T*_2_ were characterized. Additionally, two-dimensional (2D) measurements were conducted, for which EXSY *T*_2_-*T*_2_ and COSY *T*_1_-*T*_2_ exchange maps were obtained. The characterization was complemented with FT-IR spectra measurements, and the nanofiber morphology was observed using SEM. As a novelty, machine learning methods, including artificial neural networks (ANNs), were applied to characterize the local structural order of the produced nanofibers. In this study, it was shown that the nanofiber nonwoven materials made from PVA are characterized by a degree of order in the range of 0.27 to 0.61, which are more ordered than the nanofibers made from chitosan and fish gelatin, characterized by an order degree ranging from 0.051 to 0.312, where 0 represents the completely unordered network and 1 a fully ordered fabric.

## 1. Introduction

Lately, nanofibers obtained through the electrospinning process have found increasing applications, being widely used in fields such as biomedicine, where they support tissue development and regeneration, wound healing, drug delivery, air filtration, and purification, as well as in sensors and other ongoing research [1,2,3]. Among the various techniques for nanofiber production, electrospinning has become an increasingly popular method for obtaining nanofibers from different types of synthetic polymers as well as natural or synthetic biopolymers [4]. The electrospinning process applies a high voltage to generate a thin liquid jet, which is subsequently stretched and solidified into long and continuous fibers on a collector. Electrospun fibers offer several advantages due to their unique nanofiber structure, which gives them an extremely high surface area and allows the electrospinning technique to have precise control over the fiber diameter. The use of different polymer compositions and active substances can be useful in various medical applications [5,6,7,8].

In the electrospinning process, a wide range of materials can be produced in the form of nanometer or micrometer fibers, especially polymers. Natural polymers, such as chitosan, collagen [9], and fish-derived gelatin, benefit from biological recognition capacity and high bioactivity potential [10,11]. Chitosan stands out for its unique combination of biocompatibility, antimicrobial activity, and hemostatic properties, which help wound healing and tissue regeneration [12,13]. Chitosan is derived from chitin through a process called deacetylation, which is found in the exoskeletons of crustaceans such as shrimp and crabs, as well as in the cell walls of fungi [14,15]. Collagen is the main component of connective tissue and is the most abundant protein, accounting for 25–35% of total body protein [10]. Collagen has been widely used for tissue-engineering scaffolding, owing to a wealth of merits, such as natural origin, non-immunogenicity, excellent biocompatibility, and biodegradability [16]. A recent review paper, by de Farias et al., describes the recent trends in gelatin electrospun nanofibers. They show that as a natural polymer, gelatin stands out due to its abundance, bioactive properties, and affordability [17]. This is a heterogeneous mixture of polypeptides, which are derived from the controlled hydrolysis of collagen. Industrial production of gelatin typically involves two stages: a pre-treatment using acids, bases, or a combination of both, followed by thermal extraction [17,18,19]. For a temperature in the range of 25–35 °C, gelatin, which is strongly influenced by its amino acid composition and molecular weight, has the ability to partially reconstitute collagen-like triple-helix structures, forming in this way junction zones that enhance intramolecular and intermolecular interactions. Above this temperature, the helices dissociate, and the gelatin in solution is characterized by a random coil conformation. In fish gelatin, especially from those originating from cold-water species, proline and hydroxyproline are present at lower levels and can restrict chain flexibility but also stabilize the collagen-like triple helices through hydrogen bonding, enhancing junction zone formation and increasing the gel strength [17,20,21].

The use of naturally derived polymers in the electrospinning process also has certain disadvantages, namely limited mechanical properties, rapid biodegradability, and lack of consistency [22,23]. On the other hand, synthetic polymers, such as polyvinyl alcohol (PVA) and polyethylene glycol (PEG), are appreciated for their efficiency due to their superior mechanical properties, well-organized microstructure, and controlled degradation rate [24]. Despite their good biodegradability and biocompatibility, synthetic polymers do not possess biological adhesion sites [25,26,27] and pH control during cellular growth [28,29]. PVA is a water-soluble semi-crystalline polymer with attractive chemical and biological features, making it suitable for different biomedical applications [30]. In the context of wound management, PVA dressings exhibit non-adherent characteristics, minimizing the risk of trauma during removal [31]. Incorporating PVA into chitosan also increases the mechanical characteristics of the obtained dressing by creating intermolecular and intramolecular hydrogen bonds and overcoming the limitations of the dressing [32,33]. Poly(ethylene glycol) (PEG) is an ideal polymer to improve the wetting properties of nanofibers due to its hydrophilicity, solubility in water and organic solvents, and biocompatibility [34,35,36].

Nuclear magnetic resonance (NMR) is a powerful method for characterizing materials, especially polymers. One of the capabilities of nuclear magnetic resonance is that several types of pulse sequences can be used to measure different NMR parameters specific to the samples being studied [37,38,39,40]. Moreover, the 1D or 2D NMR measurements findings may be substantially enhanced by a modern analysis, which implies the use of inverse Laplace transform to obtain, not a single and global value for an NMR parameter, but the corresponding 1D or 2D distributions. Fourier transform infrared spectroscopy (FT-IR) is a modern method capable of identifying the structure of simple compounds or, if the measured sample is more complex, providing detailed information about the types of chemical bonds [41,42]. Scanning electron microscopy (SEM) and X-ray diffraction (XRD) methods can provide information about structural organization at the nano- to micrometer level [43,44]. One of the classic methods of characterizing nanofibers is performed using scanning electron microscopy (SEM). SEM provides sufficient resolution at multiple magnifications for the structural characterization of nanofibers obtained by electrospinning.

Today, artificial neural networks (ANNs) are widely used in areas like healthcare, transportation, and communication, and in many other fields, playing an increasingly important role in shaping modern society. ANNs can be developed and trained to characterize the physical and morphological properties of electrospun films. Due to its efficiency, in our study, the introduction of artificial intelligence offered clear advantages in predicting the degree of fiber alignment. ANN can also be used to detect and analyze specific issues in the structure of materials. Moreover, it allows for easier and faster adjustments of fabrication parameters [45,46,47,48].

Despite extensive studies on electrospun nanofibers, challenges remain in correlating fiber morphology and dynamics with functional properties. The major objective of the study is to use advanced methods to characterize nanofiber nonwovens produced by electrospinning, such as 1D and 2D NMR relaxometry, NMR and FT-IR spectroscopy, XRD, and SEM. This paper presents five sets of samples based on chitosan, fish gelatin, collagen derived from marine fish, PVA, and PEG, which were mixed and produced in different ratios (see Table 1). These have been chosen as some representative natural and synthetic polymers, of which the scaffolds’ properties and performance have been widely reported in the literature. Furthermore, an artificial neural network (ANN) was developed and trained to assess the degree of nanofiber alignment/order using SEM images. To our knowledge, only limited studies have applied artificial intelligence (e.g., machine learning) for the quantitative assessment of nanofiber ordering. Thus, this work not only combines classical characterization methods with modern artificial intelligence approaches but also provides comparative insights into natural versus synthetic polymer blends. To justify the choice of selected methods used in this work, a statistical analysis was employed on the number of publications related to the characterization of materials obtained by electrospinning. It was found that, from 2020 to present, the total number of publications dealing with this subject is ~12,500 (±1500). Among these, 85–95% publications used SEM as the standard method for studying electrospun nanofibers. Thus, SEM can be considered as a classical method of characterization. About 50 ± 10% papers used FT-IR spectroscopy. Despite the widespread use of nuclear magnetic resonance for material characterization, just a small number (40–100, representing 0.3–0.8%) of papers reported the occasional use of solid-state high-resolution NMR for the characterization of various materials obtained by electrospinning. In total, 15–50 papers (representing 0.1–0.4%) reported low-field NMR among the methods used for characterizing electrospun, nonwoven, nanofiber materials. Extremely rarely (only 3–10 papers, representing ~0.02–0.08%), 2D NMR techniques were used to characterize these materials, despite their many biomedical applications. Then, the statistical search was extended to the use of any kind of machine learning to characterize the electrospun nanofibers. The results show that approximately 300–400 papers (with increasing impact) used ANNs mainly to predict fiber diameter from process parameters, while only 30–80 papers (0.25–0.6%) combined ANN/ML with SEM images, usually for image classification or defect detection. None of these studies used ANN to predict the order degree.

## 2. Materials and Methods

### 2.1. Materials

The peptide collagen (land-based) was produced by Arkure Health Care (Rohtak, India). The marine fish collagen was purchased from MyProtein (Romania). Chitosan (5–20 mPa s 0.5% in 0.5 M Acetic Acid at 20 °C), PVA powder (87–89% hydrolyzed), polyethylene glycol PEG 8000, and pure glacial acetic acid (C_2_H_4_O_2_ 60.05 g/mol) were purchased from Nordic (Cluj-Napoca, Romania). Fish gelatin was produced by Louis Francois (France).

### 2.2. Electrospinning

The bio-polymeric solutions were placed into a 50 mL syringe with a needle of 0.8 mm inner diameter. The high-voltage power supply (12 kV) was connected to the needle through a conductive clamp. The feed rates of the solution were optimized. A piece of aluminum foil was fixed with adhesive tape (Tesa^®^ film) on the rotary collector to serve as the grounded collector. This was placed at a 100 mm distance at the same height as the needle. The feed rates of the solutions were optimized between 0.8 and 3.2 mL/h. Therefore, the total electrospinning deposition time ranged between approximately 15 and 62 h.

### 2.3. X-Ray Diffraction

Among the most used methods for non-destructive investigation of materials at the microscopic level is X-Ray Diffraction (XRD). X-ray radiation has wavelengths between 0.5 and 500 Å, having energies between 1 and 100 KeV, which gives it a high penetrating power [49]. To characterize the order degree of various phases in the analyzed samples, powder diffraction was used. The XRD analysis was performed at room temperature using an XRD-6000 SHIMADZU diffractometer (Kyoto, Japan), with a monochromator of graphite for the Cu-Kα radiation (λ = 1.54056 Å), at 40 kV and 30 mA, with a speed of 2°/minute and a step of 0.02° [50].

### 2.4. ^1^H NMR Relaxometry

The ^1^H NMR relaxometry measurement was performed using a Bruker Minispec MQ 20 spectrometer (Bruker BioSpin MRI GmbH, Ettlingen, Germany) working at 19.69 MHz. The NMR T2 relaxation data were recorded using the classical CPMG pulse sequence with echo times (TE) of 70 μs and 500 μs (0.5 ms). The experimental data were processed using a fast inverse Laplace-like transform (ILT) algorithm, developed to analyze multi-exponential decay curves [40].

### 2.5. ^1^H NMR Spectroscopy

For the high-field localized ^1^H NMR spectroscopy, the NMR investigation was performed using a Bruker BioSpec 70/16USR scanner (Bruker BioSpin MRI GmbH, Ettlingen, Germany) in a static magnetic field of 7.04 Tesla. The BGS 9S HP gradient unit can produce a maximum gradient of 760 mT/m. Experimentally, a volume coil with a diameter of 60 mm was used. ParaVision 5.1 software was used for data acquisition, and TopSpin 3.6.5 software was used for spectra processing. After the transmission of an excitation pulse with a duration of 5 µs, 4096 points were acquired with a dwell time (DW) of 124.8 µs. For all samples, the water peak was experimentally suppressed by choosing this option in the pulse program settings. In order to increase the signal-to-noise ratio (SNR), the localized volume was 0.83 cm^3^.

### 2.6. FT-IR Spectroscopy

The FT-IR spectra were measured using a Jasco 6200 FT-IR spectrometer (Ishikawamachi Hachiojishi, Tokyo, Japan). For electrospinning solutions and nanofibers production, 10–15 mg of samples were mixed with 200 mg of KBr powder in an agate mortar. The resulting powder was transferred into a mold and then pressed at 15 mT, resulting in a 10 mm diameter tablet, which can be placed into a holder inside the FT-IR spectrometer. For background, a simple KBr tablet was used. The spectrometer parameters were set as follows: the wavenumber $\tilde{\nu }$ was fixed between 349.053 cm^−1^ and 4000.6 cm^−1^, 64 scans were accumulated in order to increase the signal-to-noise ratio (SNR), the resolution was 4 cm^−1^, a zero-filling followed by a cosine apodization procedure was performed, the auto filter was 10 kHz, and the scanning speed was 2 mm/s.

### 2.7. Scanning Electron Microscopy

The structures of the electrospun fibers were observed using JEOL JSM 5600LV, a scanning electron microscope (SEM) (Akishima, Tokyo, Japan). All images were obtained at 15 kV accelerating voltage, using the secondary electron signal. Prior to analysis, the samples were gold-coated using a sputtering device. By using the secondary electron signal, the morphology of the samples is analyzed, with details of the sample surface being recorded. The aspect of the fibers’ lengths and widths can be visualized and measured to understand the deposition and structure formation of the fiber films.

### 2.8. Artificial Neural Network Analysis

An artificial neural network (ANN) can be defined simply as a numerical architecture for machine learning through examples. Such a virtual machine, which can be trained to learn, can take on complex tasks and, eventually, become capable of solving problems for which it is difficult to provide a simple solution. They can also quickly process large amounts of data and provide real-time predictions [51,52,53]. ANN models are implemented worldwide for image recognition, posture recognition, and speech recognition, among other applications. They are increasingly applied in various scientific domains. Recently, applications have been reported for quantitative and qualitative analyses in Fourier spectroscopy [54,55] and in Laplace spectroscopy [52].

## 3. Results and Discussions

### 3.1. Characterization of Raw Materials

#### 3.1.1. Crystallin to Amorphous Character Evaluated from XRD Characterization

**Figure 1:**
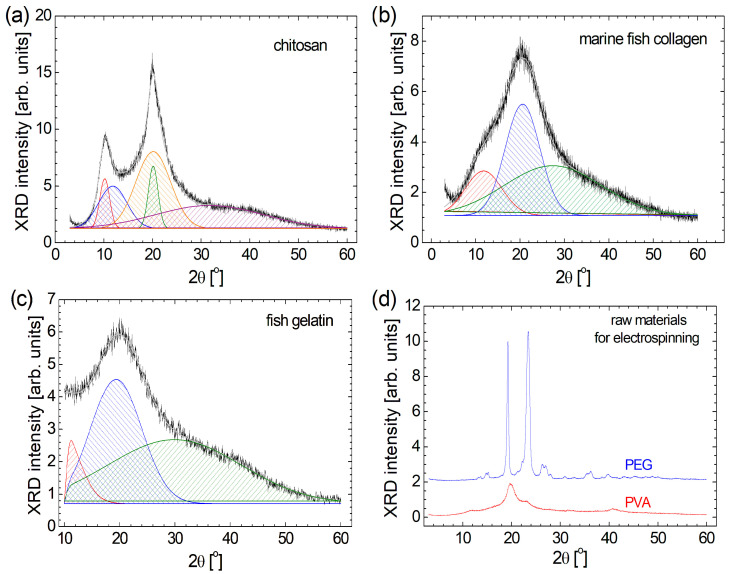
The deconvolution of XRD patterns recorded for (**a**) chitosan, (**b**) marine fish-collagen, (**c**) fish gelatin, and (**d**) XRD patterns corresponding to PEG and PVA.

From a geometrical and macromolecular arrangement point of view, the biomaterials such as chitosan, marine fish collagen, and fish gelatin present a semi-crystalline structure [1,4]; this is confirmed from the relatively broad peak observed in the XRD patterns presented in Figure 1. Conversely, organic polymers such as PEG and PVA present a more crystalline structure. For a better description of semi-crystalline raw materials, their XRD diffractograms have been deconvoluted. In this sense, one can observe that all three raw biomaterials present a very broad peak that appears at large 2θ angles: ~31.7° for chitosan (Figure 1a), ~27.4° for marine fish collagen (Figure 1b), and ~30.0° for fish gelatin (Figure 1c). These components are so broad that they cover the diffraction angles from below 10° up to 60°, representing 33.1% of chitosan, 63.9% of marine fish collagen, and 47.4% of fish gelatin. The most intense and relatively broad peak observed at 2θ = 19°–21° can be correlated with the diameter of the left-handed triple-helical if one can speak about the collagen molecule, as in the case of marine fish collagen (2θ ≅ 20.6°) and partially hydrolyzed as in the case of fish gelatin (2θ ≅ 19.4°) [2]. In the case of chitosan, one can also observe a broad peak located at 2θ ≅ 20.2°, having a similar source but also a narrow peak almost in the same position (2θ ≅ 20.1°). A diffraction angle 2θ below 12° can reflect the diameter of the triple-helix structure of the protein [2]. Again, in the case of marine fish collagen and fish gelatin, one can observe a single relatively broad peak centered at 2θ ≅ 11.8° and 11.2°, respectively. In the case of chitosan, a similar peak appears at 2θ ≅ 11.9°, but it is accompanied by another peak centered at 2θ ≅ 10.2°. In a relatively recent study, the deconvolution of the XRD pattern recorded for chitosan with different molecular weights was also deconvoluted using different methods. The one, named the peak deconvolution method, provided similar results, presenting two narrow, crystalline peaks located at 2θ ≅ 10° and 2θ ≅ 20°, and two amorphous, wide peaks [56]. Therefore, the marine fish collagen and fish gelatin exhibit a similar XRD pattern, which can also be observed for chitosan, but with two additional narrow peaks at 2θ ≅ 10.2° and 20.2°, possibly explaining the greater mechanical hardness of this biomaterial. Similarly, XRD patterns are reported by Mo et al. [57] for acid-soluble collagen (ASC) and pepsin-soluble collagen (PSC) samples from the Swim bladders of *Megalonibea fusca* and by de Farias et al. [58] for gelatin powder with different origins: bovine, porcine, and fish.

Among the raw materials used for this study, PEG is the most crystalline. One can remark two narrow peaks with high amplitude at 2θ ≅ 19.3° and 23.4° (see Figure 1d). Barron et al. reported a similar XRD pattern for PEG 8000 with the two narrow peaks located at 2θ ≅ 19.25° and 23.4° [59]. For the PVA sample, the highest two peaks can also be found in approximately the same positions (2θ ≅19.8° and 22.9°), but their amplitudes are much lower compared to the amplitude of the highest peak observed in the XRD pattern of PEG. With the exception of the peak located at 2θ ≅ 11.5°, the XRD pattern (Figure 1d), which is not so visible, looks similar to that reported by Aziz et al. [60]. For the PEG raw material, a series of doublets can also be observed (at 2θ ≅ 13.1° and 13.7°, at 2θ ≅ 14.8° and 15.3°, at 2θ ≅ 26.3° and 27.0°, and at 2θ ≅ 35.5° and 36.3°), reflecting a repetitive and complex structure. The PVA is simpler, and the small amplitude peaks are relatively broad. This fact indicates that PVA is more amorphous than PEG.

#### 3.1.2. Structural and Dynamics Characterization by FT-IR Spectroscopy and ^1^H NMR Relaxometry

**Figure 2:**
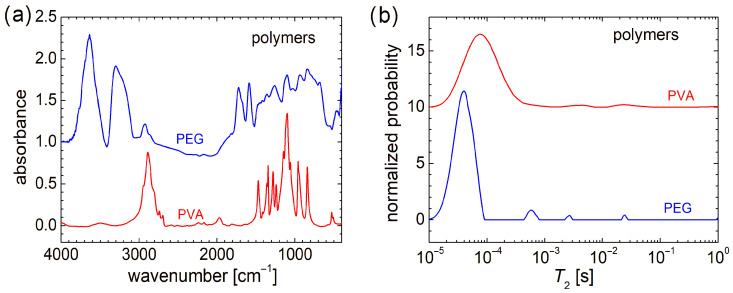
(**a**) FT-IR spectra and (**b**) distribution of transverse relaxation times (*T*_2_) measured for PEG and PVA polymers.

From a molecular and structural point of view, the FT-IR spectra indicate that PEG (or HO[CH_2_CH_2_O]_n_H) and PVA ([CH_2_CH(OH)]_n_), despite the similarity in chemical formula, present large variations (see Figure 2a). The FT-IR spectra recorded for chitosan, marine fish collagen, and fish gelatin are presented in Ref. [4] and largely discussed; therefore, they are not shown here again. Compared with the FT-IR spectra of raw biomaterials, the FT-IR spectra of PEG and PVA are more well resolved (especially that of PVA). While the FT-IR spectrum of fish gelatin consists of 2–3 broad bands, the FT-IR spectrum of marine collagen is similar to that of fish gelatin, with a slightly better-defined structure [4]. In this sense, one can mention the peaks located at ~1652 cm^−1^ (associated with the stretching vibrations of carbonyl groups, C=O double bond from amide I [61]) and ~1540 cm^−1^ (associated with the bending vibration of N–H bond coupled with the stretching vibration of C–N bond [61,62]), which may have a correspondent in the chitosan FT-IR spectrum and are relatively close to some narrow bands of PEG (~1589 cm^−1^ and ~1722 cm^−1^). The differences between FT-IR spectra of raw biopolymers and PEG and PVA are natural. The absorption lines of the latter two can have the origin only in the vibration of O-H and C-H bonds, and symmetric and asymmetric stretching of the CH_2_ functional group (and of the molecular backbone). The similarities between the FT-IR spectra of studied organic polymers (PEG and PVA) and those recorded for raw biopolymers can be summarized as follows: (i) in the range between ~3000 cm^−1^ and 3800 cm^−1^ for PEG and chitosan; (ii) in the range of ~2800 cm^−1^ and 3000 cm^−1^ for PVA and chitosan and, to a lesser extent, PEG; (iii) the high and narrow peak located at ~1100 cm^−1^ for PVA and chitosan. A detailed description of FT-IR spectra recorded for PEG and PVA can be found in Refs. [63,64].

From a molecular dynamics point of view, as can be observed from the transverse relaxation time, *T*_2_-distributions presented in Figure 2b, PEG and PVA are more similar to each other and also to chitosan, marine fish collagen, and fish gelatin (of which *T*_2_-distributions are presented in Ref. [4]). In this sense, PEG presents a major peak located at a *T*_2_-value of ~40 µs, which is the smallest one compared with PVA, whose major peak is located at *T*_2_ ≅ 76 µs (see Figure 2b), and *T*_2_ ≅ 115 µs measured for marine fish collagen, *T*_2_ ≅ 164 µs measured for chitosan, and *T*_2_ ≅ 240 µs measured for fish gelatin [4]. This could be an explanation of the mechanical hardness exhibited by PVA and PEG samples compared to raw biomaterials. All samples present a series of well-resolved or broad peaks, characterized by a small integral area (i.e., a small amount of ^1^H) located at higher *T*_2_-values and indicating more mobile components. This can originate from long polymeric chains or dangling/loose ends. Thus, PEG presents a series of three well-resolved peaks located at ~540 µs, ~2.68 ms, and ~24.1 ms. The last two peaks are in the range where two peaks are observed for chitosan and fish gelatin, which also present well-resolved peaks. In the *T*_2_-distribution measured for PVA, two broad peaks are observed. This *T*_2_-distribution resembles more closely that measured for marine fish collagen [4]. Both types of samples, organic polymers (PEG and PVA) or biopolymers, used as raw materials for electrospinning, present the largest amount of ^1^H within extremely rigid components (with reduced molecular mobility) but also contain some dynamic components (two or three) with increased mobility, although in much lower amounts.

### 3.2. Characterization of Electrospun Solutions

#### 3.2.1. Complex Interactions in Electrospun Solutions Evaluated from High-Field ^1^H NMR Spectroscopy

**Figure 3:**
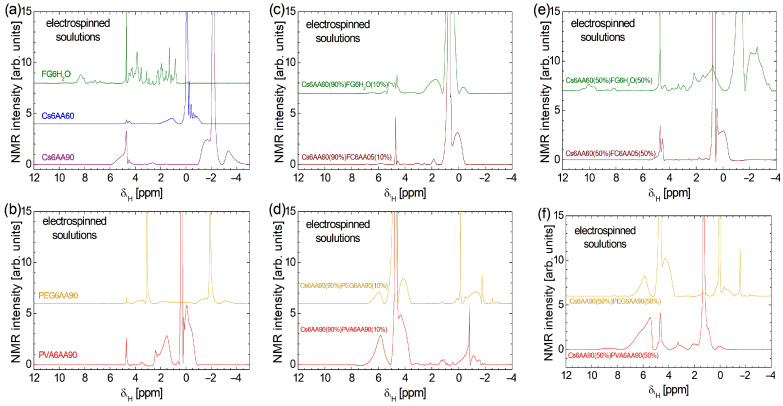
High-field ^1^H NMR spectra measured (**a**) for pure solution of acetic acid (90% or 60% and distillated water) and 6% *w*/*v* chitosan and fish gelatin and (**b**) for pure solution of 90% acetic acid and 6% *w*/*v* PVA or PEG; (**c**) for 90% solution 1 of 6% *w*/*v* chitosan (AA 60%) and 10% of solution 2 6% *w*/*v* marine fish collagen (AA 5%), fish gelatin (100% distillated water); (**d**) for 90% solution 1 of 6% *w*/*v* chitosan (AA 90%) and 10% of solution 2 of 6% *w*/*v* PVA (AA 90%) and PEG (AA 90%); (**e**) for 50% solution 1 of 6% *w*/*v* chitosan (AA 60%) and 50% of solution 2 of 6% *w*/*v* marine fish collagen (AA 5%), fish gelatin (100% distillated water); (**f**) for 50% solution 1 of 6% *w*/*v* chitosan (AA 90%) and 50% of solution 2 of 6% *w*/*v* PVA (AA 90%), and PEG (AA 90%).

An excessively high concentration of acetic acid may lead to collagen denaturation. This effect was previously extensively discussed in Refs. [1,3,4]. It was found that an amount of 10–20% acetic acid (AA) may start to produce collagen denaturation, which prevents fiber formation if a simple collagen with acetic acid solution is used [4]. Nevertheless, if the solution is combined with another one (e.g., chitosan-based), then nanofiber films may be obtained. The effect of acetic acid concentration on collagen can be easily observed by comparing the high-field localized ^1^H NMR spectra. These are presented at the bottom of Figure 3a for samples labeled Cs6AA90 and Cs6AA60. In the range from ${\delta_{1}}_{H}$ ≈ 4.0 ppm to ${\delta_{1}}_{H}$ ≈ 6.0 ppm, one can observe specific water-suppressed peaks. This procedure is essential in the case of large quantities of water compared with the ^1^H originating from components with a reduced content of hydrogen. One can observe that the water suppression procedure is more efficient in the case of Cs6AA60, where the water content in the acetic acid solution is higher. Then, one can observe that the acetic acid peak (the highest peak, which was cut to emphasize the components with small amplitude) appears at ${\delta_{1}}_{H}$ ≈ −0.08 ppm for Cs6AA60 solution and at ${\delta_{1}}_{H}$ ≈ −2.14 ppm for Cs6AA90 solution. This effect of displacement of the acetic acid peak was observed and discussed in Ref. [4] but, in our case, was much larger due to the increased concentration of AA. Observing the linewidth of the acetic acid peak (which is narrow), one can conclude that the specific transverse relaxation time, *T*_2_, was long, which indicated a mobile component. Then, one can say that, even in interactions with collagen, the majority of acetic acid molecules remain mobile. Other, relatively broader peaks can be associated with less mobile marine fish collagen fraction interacting with AA.

The most complex high-field ^1^H NMR spectrum was recorded for fish gelatin dissolved in distilled water (see the third, olive-colored spectrum in Figure 3a). One can observe a series of narrow, relatively well-resolved peaks, which indicate that the fish gelatin presents a large variety of components (the difference in chemical shift—${\delta_{1}}_{H}$ from ~0.71 ppm up to ~5.03 ppm—is due to differences in the local environment), all with a large mobility. A series of doublets, less well-resolved and with smaller amplitudes, are observed at the chemical shift ${\delta_{1}}_{H}$ from ~6.66 ppm up to ~7.63 ppm, while a broad line with almost unresolved peaks is observed ${\delta_{1}}_{H}$ in the range from ~7.91 ppm up to ~8.67 ppm. This may correspond to fish gelatin components with a slightly reduced mobility.

The high-field ^1^H NMR spectrum of PVA6AA90 consists of fewer components and is distributed over a narrow domain in the range of ${\delta_{1}}_{H}$ from ~−0.9 ppm up to ~4.8 ppm (see the fourth, red spectrum in Figure 3b). The most intense peak is centered at ${\delta_{1}}_{H}$ ≈ 0.37 ppm and is associated with the AA. The small linewidth indicates that this component is characterized by a large mobility, and it may correspond to a relatively free fraction of AA. A relatively broad peak is observed at ${\delta_{1}}_{H}$ ≈ −0.06 ppm, which also presents a broad shoulder at smaller ${\delta_{1}}_{H}$-values and a narrow shoulder at larger ${\delta_{1}}_{H}$-values (~0.186 ppm). This may correspond to the PVA polymer in interaction with the AA. The broad peaks indicate that the mobility of the corresponding components is reduced. There are also some other peaks, but we will mention only the broad peak located at about 1.53 ppm and the narrow peak at ${\delta_{1}}_{H}$ ≈ 2.40 ppm, as having a significant integral area and thus corresponding to components with significant content.

PEG6AA90 solution presents one of the simplest high-field ^1^H NMR spectra (see the upper orange spectrum in Figure 3b). The AA peak is found at ${\delta_{1}}_{H}$ ≈ −1.88 ppm, a location close to those found in the case of the Cs6AA90 sample. The narrow linewidth indicates that a large fraction of AA presents a large mobility. The relatively broad and small peaks located left and right of this peak indicate the fraction of AA in interaction with the PEG polymer, which has a reduced mobility. The peak located at ${\delta_{1}}_{H}$ ≈ −1.88 ppm is not the largest peak in the ^1^H NMR spectrum of the PEG6AA90 solution. Another peak located at ${\delta_{1}}_{H}$ ≈ 3.09 ppm presents the highest amplitude and is accompanied also by left (${\delta_{1}}_{H}$ ≈ 3.53 ppm) and right (${\delta_{1}}_{H}$ ≈ 1.86 ppm) wide peaks, which may be associated with the PEG polymer in interaction with the AA90 solution with a reduced mobility (and/or local heterogeneous environment).

The high-field ^1^H NMR spectra of bi-component solutions (see Figure 3b–f) are not a simple superposition of mono-component solutions (Figure 3a,b). This indicates a complex interaction between all components of the solutions, even after mixing each pair of components. Figure 3c,d present the water suppression high-field ^1^H NMR spectra recorded for the two-component electrospun solutions, where the chitosan-based solution is 90% and the second component, the polymer-based solution, is 10%. First, one can observe that the water suppression procedure is efficient for fish collagen/gelatin-based solution (i.e., Cs6AA60(90%)FC6AA05(10%) and Cs6AA60(90%)FG6H_2_O(10%)), as can be seen from the relatively small amplitude of the peaks located at ${\delta_{1}}_{H}$ ≈ 4.69 ppm. Conversely, for the solutions based on organic polymers (i.e., Cs6AA90(90%)PVA6AA90(10%) and Cs6AA90(90%)PEG6AA90(10%)), the water peak is less suppressed, indicating an additional interaction between water molecules and organic polymers, PVA and PEG. The main peak representing the most mobile AA component is displaced towards a smaller chemical shift, e.g., ${\delta_{1}}_{H}$ ≈ 0.76 ppm for Cs6AA60(90%)FC6AA05(10%) solution, ${\delta_{1}}_{H}$ ≈ 0.49 ppm for Cs6AA60(90%)FG6H_2_O(10%) solution, ${\delta_{1}}_{H}$ ≈ −0.13 ppm for Cs6AA90(90%) PEG6AA90(10%) solution, and ${\delta_{1}}_{H}$ ≈ −0.82 ppm for Cs6AA90(90%)PVA6AA90(10%) solution. The more viscous components (polymer in interaction with fluids—acetic acid/water—such as chitosan, marine fish collagen, fish gelatin, PEG, or PVA) characterized by a reduced molecular mobility are represented by the broadened peaks, which appear left and right of the main peaks centered around (i) AA peaks (with variable position in ${\delta_{1}}_{H}$ scale) and (ii) the water peak located at ${\delta_{1}}_{H}$ ≈ 4.70 ppm. The magnitude of these peaks, which are observed for Cs6AA90(90%)PVA6AA90(10%) and Cs6AA90(90%)PEG6AA90(10%) solutions in the range of ${\delta_{1}}_{H}$ from ~3.47 ppm up to ~7.23 ppm, may be an indication of a stronger interaction between PVA and PEG with water molecules, compared to the weak interaction between chitosan or fish collagen and gelatin with water.

The increase in the content of the second (non-chitosan-based) component from 10% to 50% has various effects on the high-field ^1^H NMR spectra measured for these solutions and presented in Figure 3e,f. This is a clear indication of various affinities of the components for water, acetic acid, or each other, not only initially (when the base solutions were prepared) but also in the mixing process of the two components. The less affected ^1^H NMR spectrum is those measured for Cs6AA60(50%)FC6AA05(50%). Here, one can observe a variation in some peak intensities, as in the case of peaks centered at ${\delta_{1}}_{H}$ ≈ 4.52 ppm or ${\delta_{1}}_{H}$ ≈ 0.67 ppm and a better resolution for the peak centered at ${\delta_{1}}_{H}$ ≈ 4.43 ppm. This indicates that the marine fish collagen undergoes the primary interaction from the formation of basic solutions of FC6AA05. Moderate variation with the change in component content are observed in the high-field ^1^H NMR spectra measured for Cs6AA90(50%)PEG6AA90(50%) and Cs6AA90(50%)PVA6AA90(50%), where with the increase in the second (non-chitosan-based) solution, some narrow peaks are much reduced, slightly repositioned, and/or converted into broadened peaks, indicating a moderate interaction between components after the mixing process, with the result of the decreased in mobility. From these spectra, one can conclude that after fish collagen, PEG and then PVA undergo the majority of interactions from the stage of primary solutions and also may interact in two-component mixtures. Similar to marine fish collagen, the fish gelatin dissolved in water presents the largest changes in the ^1^H NMR spectrum once the proportion of the FG6H_2_O solution increases. The spectrum becomes broadest and is observed over a chemical shift ${\delta_{1}}_{H}$ range from ~−3.69 ppm up to ~11.16 ppm. One can remark that the specific features of the FG6H_2_O solution shifted from the range from ~6.66 ppm up to ~7.63 ppm to the range from ~7.73 ppm up to ~11.16 ppm. In the range of ${\delta_{1}}_{H}$ from ~0.0 ppm up to ~5.32 ppm from well-resolved peaks (indicating a well-organized structure) are replaced by broadened peaks, indicating an interaction with the formation of viscous components characterized by a reduced mobility. Finally, one can observe the acetic-acid-specific features in the range of ${\delta_{1}}_{H}$ from ~−3.69 ppm up to ~0.0 ppm.

#### 3.2.2. Molecular Dynamics of Liquid Solutions Evaluated by Low-Field ^1^H NMR Relaxometry

**Figure 4:**
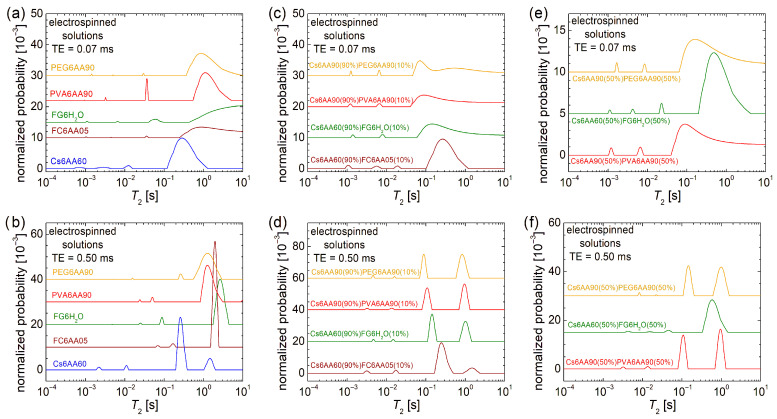
Low-field ^1^H NMR *T*_2_-distributions measured for pure solution of acetic acid (90% or 60% and distillated water) and 6% *w*/*v* chitosan, fish gelatin, PVA, and PEG for an echo time of (**a**) TE = 0.07 ms and (**b**) TE = 0.50 ms; 90% solution 1 of 6% *w*/*v* chitosan (AA 90% or 60%) and 10% of solution 2 of 6% *w*/*v* marine fish collagen (AA 5%), fish gelatin (100% distillated water), PVA (AA 90%), and PEG (AA 90%) for an echo time of (**c**) TE = 0.07 ms and (**d**) TE = 0.50 ms; 50% solution 1 of 6% *w*/*v* chitosan (AA 90% or 60%) and 50% of solution 2 of 6% *w*/*v* marine fish collagen (AA 5%), fish gelatin (100% distillated water), PVA (AA 90%), and PEG (AA 90%) for an echo time of (**e**) TE = 0.07 ms and (**f**) TE = 0.50 ms.

The overall dynamics characterization at the molecular level can be performed by low-field ^1^H NMR relaxometry. In this sense, the normalized *T*_2_-distributions measured for the electrospun solutions are presented in Figure 4. In the first row, the echo time, TE = 0.07 ms, emphasizes the low *T*_2_-values and suppresses the high *T*_2_-values, while in the second row, the TE = 0.50 ms emphasizes the high *T*_2_-values by resolving the peaks and acts as a filter for the low *T*_2_-values. Both of them offer the complete view, but for the liquid samples, as they are the measured solutions, the majority of dynamic components are characterized mainly by high *T*_2_-values; therefore, our focus is on the second row in Figure 4.

In the case of the one-component solutions, one can observe that the most viscous solution, i.e., Ch6AA60, presents the main peak (the largest integral area) located at *T*_2_ ≈ 262 ms, while the PVA6AA90 solution presents the main peak located at *T*_2_ ≈ 1.241 s value, which is close to that obtained for the PEG6AA90 solution (*T*_2_ ≈ 1.271 s) but smaller compared with the *T*_2_ ≈ 1.951 s measured for the FC6AA05 solution, which is smaller compared with the *T*_2_ ≈ 2.617 s measured for the FG6H_2_O solution, which contains no acetic acid. All *T*_2_-distributions are characterized by two peaks located at smaller *T*_2_-values, indicating the presence in all samples of some undissolved bio or organic polymeric components of different sizes and moderate mobility.

Examining the *T*_2_-distribution measured for the Cs6AA60 solution, one can also observe a peak with significant integral area (second after the main peak) located at *T*_2_ ≈ 1.455 s, which also indicates the presence of a component with high mobility. Nevertheless, the *center of gravity* of the *T*_2_-distribution measured for the Cs6AA60 solution converges towards the smaller *T*_2_-values, thus indicating a reduced mobility or, vice versa, an increased viscosity. This high viscosity makes the chitosan-based solutions create problems at electrospinning due to the difficulty of compressing the solution through the needle. The *solution* to the problem is the use of a chitosan-based solution mixed with other polymer solutions to decrease the overall viscosity. Additionally, any debris that can clog the needle, i.e., undissolved polymeric fractions, may affect nanofiber production. Their mobility is inversely proportional to their size, and therefore, the largest undissolved polymeric fractions are associated with the smallest *T*_2_-values. And, as one can observe from Figure 4a, all solutions are characterized by even low-mobility components. These are associated with the peaks located under 1 ms in the *T*_2_-distributions presented in Figure 4a and among all samples; Cs6AA60 solution presents the largest integral area for this peak.

The two-component mixtures of chitosan-based solutions in 90% and bio- and organic-polymeric-based solutions in 10% present interesting and similar *T*_2_-distributions (see Figure 4d). These consist of a main doublet located at large *T*_2_-values (≥89.1 ms) and another minor doublet located at medium *T*_2_-values (between 2.6 ms and 20.0 ms). This indicates that the major dynamical behavior is dictated by the large content of Cs6AA60 solution. The types of bio- or organic polymers have the largest influence on the position of the left peak of the main doublet, i.e., *T*_2_ ≈ 90.17 ms for Cs6AA90(90%)PEG6AA90(10%) solution, *T*_2_ ≈ 108.4 ms for the Cs6AA90(90%)PVA6AA90(10%) solution, *T*_2_ ≈ 143.7 ms for the Cs6AA60(90%)FG6H_2_O(10%) solution, and *T*_2_ ≈ 249.1 ms for the Cs6AA60(90%)FC6AA05(10%) solution. With the exception of the solution containing marine fish collagen, the rest of three solutions present the *right* peak from the main doublet almost at the same position and with an integral area comparable to that of the *left* peak, indicating that the most mobile component is less affected by the type of polymer and most probably originates from the most abundant solution, the component one, i.e., Cs6AA90(90%). The Cs6AA60(90%)FC6AA05(10%) solution presents the most mobile components, as indicated by the presence of the peak located at *T*_2_ ≈ 1489 ms, but the contribution of this one is significantly less than the *left* peak. The minor doublets are almost in the same position, separated by an order of magnitude of *T*_2_-values, indicating similar dynamic behavior of small amounts of polymers. Additionally, the short TE measurements (see Figure 4c) show that only the Cs6AA60(90%)FC6AA05(10%) solution presents three minor peaks in the measured *T*_2_-distribution.

A balanced ratio (1:1) between the two-component solutions will not change much in the dynamic behavior for the solutions based on organic polymers PEG and PVA (i.e., Cs6AA90(50%)PEG6AA90(50%) and Cs6AA90(50%)PVA6AA90(50%)) compared with the case of unbalanced content (9:1) discussed above, as can be seen from Figure 4f. The most stable dynamics are observed for the Cs6AA90(50%)PVA6AA90(50%) solution, while the *T*_2_-distribution measured for Cs6AA90(50%)PEG6AA90(50%) solution presents a displacement of peaks towards larger *T*_2_-values. This fact indicates that the addition of a larger proportion of PEG6AA90 slightly increases the mobility of the final solution. The most spectacular increase in mobility was obtained for the solution containing fish gelatin. Thus, in this case, the *left* peak from the main doublet shifts from *T*_2_ ≈ 249.1 ms (see Figure 4d) to *T*_2_ ≈ 583.4 ms. But the most spectacular displacement can be observed for the *right* peak from the main doublet, which presents a shift from *T*_2_ ≈ 1.021 s (see Figure 4d) to a *T*_2_-value larger than 10 s (see the cut peak located at the highest *T*_2_-values in Figure 4f). This is a clear indication that the increase in the FG6H_2_O content (proportion) leads to a dramatic increase in mobility, which was also observed as a dramatic decrease in viscosity, which, at the limit, may be an important factor that may lead to an inability to obtain nanofibers by electrospinning. Using the Cs6AA60(50%)FG6H_2_O(50%) solution, it was possible to obtain nanofibers (as will be shown below), but we could not obtain nanofibers by electrospinning using the Cs6AA60(50%)FC6AA05(50%) based on marine fish collagen, and therefore, the corresponding *T*_2_-distribution was not measured for this solution. All of these solutions are also characterized by undissolved components with reduced molecular dynamics. Figure 4e better represents the position and the integral area of these peaks associated with reduced dynamics components, which are more immobile and with a higher content, as can be inferred from Figure 4f.

#### 3.2.3. FT-IR Spectroscopy of Mono- and Two-Component Electrospun Solutions

**Figure 5:**
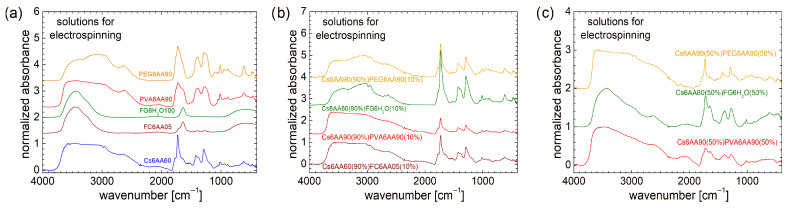
FT-IR spectra measured for (**a**) pure solution of acetic acid (90% or 60% and distillated water) and 6% *w*/*v* chitosan, fish gelatin, PVA, and PEG; (**b**) 90% solution 1 of 6% *w*/*v* chitosan (AA 90% or 60%) and 10% of solution 2 of 6% *w*/*v* marine fish collagen (AA 5%), fish gelatin (100% distillated water), PVA (AA 90%), and PEG (AA 90%); (**c**) 50% solution 1 of 6% *w*/*v* chitosan (AA 90% or 60%) and 50% of solution 2 of 6% *w*/*v* marine fish collagen (AA 5%), fish gelatin (100% distillated water), PVA (AA 90%), and PEG (AA 90%).

Chitosan, PVA, and PEG dissolved in a solution of AA 90% present a more detailed structure compared to marine fish collagen dissolved in AA 05% and fish gelatin dissolved in H_2_O, as can be seen from the FT-IR spectra measured for these solutions and represented in Figure 5a. Compared to the FT-IR spectra measured for the raw PEG and PVA, which present large differences (see Figure 2a), the dissolution of these two organic polymers in a concentrated solution of acetic acid (90%) leads to a *homogenization* of features from FT-IR spectra, which may be explained only by a strong influence of acetic acid on the final structure of the prepared solutions. The only significant difference between the FT-IR spectra recorded for PEG6AA90 and PVA6AA90 can be found in the band from 2800 cm^−1^ to 3400 cm^−1^, and this is most probably due to the absorption line of PEG from 3296 cm^−1^ (see Figure 2a). A concentrated solution of acetic acid (90%) leads to a relatively similar but better resolved FT-IR spectrum, for the case of Cs6AA90 solution. The large amount of water (100% in the case of FG6H_2_O and 95% in the case of FC6AA05) also has a great influence on the FT-IR spectra of these solutions, which present a typical liquid spectrum with only small influences from marine fish collagen and fish gelatin.

It is not a surprise that all FT-IR spectra recorded for the unbalanced (90%/10%) two-component solutions resemble the FT-IR spectrum recorded for the Cs6AA90 solution more. The larger resemblances between the FT-IR spectrum of a two-component solution with the FT-IR spectrum of a one-component Cs6AA90 solution are found in the case of Cs6AA60(90%)FC6AA05(10%). Then, one can say that the marine fish collagen dissolved in AA 5% in a small amount made an insignificant contribution to the chemical bonding of the final two-component solutions. PVA is a hard polymer, and therefore, it is surprising that PVA6AA90 has only a small influence on the two-component Cs6AA90(90%)PVA6AA90(10%) solution structure. The corresponding FT-IR spectra resemble those of Cs6AA90 more, especially in the range of large wavenumbers (≥1850 cm^−1^), while in the range of small wavenumbers (<1850 cm^−1^), the spectra preserve the structure, but the absorbance is lower.

The FT-IR spectrum recorded for Cs6AA60(90%)FG6H_2_O(10%) solution resembles more the spectrum recorded for Cs6AA90(90%)PEG6AA90(10%) solution, especially in the range of 2500 cm^−1^ to 3350 cm^−1^, where the fish gelatin displays the specific features that were observed also in the case of PEG6AA90 mono-component solution (see Figure 5a). In the range of low wavenumbers (<1850 cm^−1^), the specific features more closely resemble those observed for Cs6AA60 mono-component and Cs6AA60(90%)FC6AA05(10%) two-component solutions, with the exception that the absorbance is higher, especially in the case of Cs6AA60(90%)FG6H_2_O(10%) solution.

At first glance, the increased content of the second component (non-chitosan-based) from 10% to 50% presents a surprising effect on the two-component solutions used for electrospinning, as can be seen from the FT-IR spectra presented in Figure 5c. If the expectation was to observe some enhanced features due to the presence of PVA, PEG, or fish gelatin, the measured spectra present, conversely, a series of attenuated features. Thus, all peaks located in the range of low wavenumbers (<1850 cm^−1^) present a reduced absorbance and broadened linewidths, and therefore, are not so well resolved. This is a clear indication that the components continue to interact and to form a more heterogeneous environment while retaining some base features. The most heterogeneous solution (among the studied ones) is Cs6AA90(50%)PVA6AA90(50%). This fact is not surprising since, as was presented above, the Cs6AA90(90%)PVA6AA90(10%) two-component solution had the smallest absorbance in the range of low wavenumbers. Fish-gelatin-based solution (i.e., Cs6AA60(50%)FG6H_2_O(50%)) presents a certain degree of heterogeneity, associated with the medium interaction between components after mixing in balanced proportions, but also preserves the specific features relatively well. The only two-component solution that may present some narrow lines in the measured FT-IR spectrum is Cs6AA90(50%)PEG6AA90(50%). This is an indication that there is still a portion of Cs6AA90 left that has not fully interacted with the PEG6AA90 components. Conversely, one can say that, at the same content, PEG6AA90 only partially *consumes* the Cs6AA90 solution, while FG6H_2_O *consumes* more, and the largest consumption is performed by the PVA6AA90 solution. For large wavenumbers, one can observe that the specific *hump* observed in the FT-IR spectra recorded for the one-component PEG6AA90 (see Figure 5a) and two-component Cs6AA90(90%)PEG6AA90(10%) and Cs6AA60(90%)FG6H_2_O(10%) (see Figure 5b), in the range of 2735 cm^−1^ to 3345 cm^−1^, is well attenuated for the balanced (50:50) two-component solutions. This is an indication that the changes also occur at the function group level.

### 3.3. Characterization of Electrospun Nonwoven Films

#### 3.3.1. Surface Area, Porosity, and Nanofibers’ Network Hierarchy Evaluated by SEM

**Figure 6:**
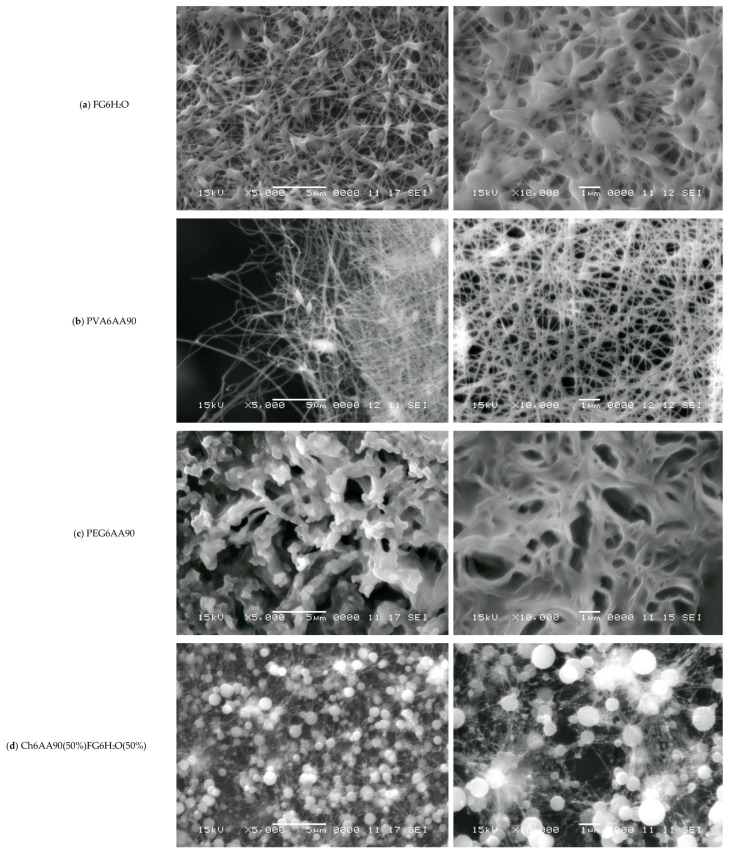

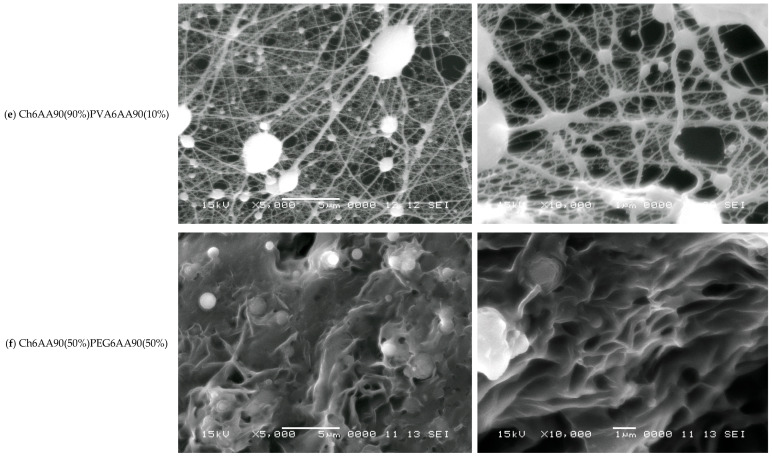
SEM images with the magnification ×5000 (**left**) and ×10,000 (**right**) measured for (**a**) FG6H_2_O; (**b**) PVA6AA90; (**c**) PEG6AA90; (**d**) Ch6AA90(50%)FG6H_2_O(50%); (**e**) Ch6AA90(90%)PVA6AA90(10%); (**f**) Ch6AA90(50%)PEG6AA90(50%). The horizontal white scale bars represent a distance of 5 μm for all SEM images with ×5000 magnification and a distance of 1 μm for all SEM images with ×10,000 magnification.

One of the classical methods used to characterize nanofibers is SEM. The resolution at different magnifications of SEM images is more than adequate for the structural characterization of nanofibers obtained by electrospinning. In Figure 6 are presented a series of SEM images at two magnifications, three for the one-component (FG6H_2_O, PVA6AA90, and PEG6AA90) and three for the balanced two-component (Ch6AA90(50%)FG6H_2_O(50%), Ch6AA90(90%)PVA6AA90(10%), and Ch6AA90(50%)PEG6AA90(50%) nonwoven films). As is known, fish gelatin is a good material that can be easily dissolved in water and which can then form a dense structure of nanofibers. The SEM micrographs of FG6H_2_O are presented in Figure 6a, with magnifications of ×5000 in the left-side image and ×10,000 in the right-side image. One can observe that the produced fibers are short, with lengths ranging from 1 µm to 5 µm, with a variable thickness of the order of several hundreds of nanometers. These fibers are connected to a series of two-dimensional centers of the order 1–2 µm with a certain thickness. These are closely packed in multiple layers of deposition during the electrospinning. The overall impression is that the electrospun FG6H_2_O bio-nanofibers are at the boundary between a nanofiber network and a porous material. Conversely, the organic-polymer-based PVA6AA90 presents a pure fibrillary network (see Figure 6b), while the organic-polymer-based PEG6AA90 presents an obvious porous material structure (see Figure 6c).

Polyvinyl alcohol in a solution of concentrated acetic acid can be formed by electrospinning a classical nanofiber nonwoven material. Thus, Figure 6b-left presents the SEM micrograph of a part of the PVA6AA90 film at an edge. In the center to right side of the image, one can see a dense network of nanofibers with variable diameters (in the tens to hundreds of nm) and with a length of several tens of micrometers. Several elliptical (rotation ellipsoids—or rugby ball) formations appear, which join a series of long nanofibers. From the left side of the image, being at the edge of the film, characterized by a reduced density, the length of fibers can be estimated much better than from the crowded, dense network. The dense nanofiber network can be well observed in the right of Figure 6b. This is an image of a random network of quasi-straight nanofibers mostly chemically cross-linked with a high density of junction points.

The surface of PEG6AA90 nano-structured films looks more organic (see Figure 6c), resembling that of a coral reef. The fibers are so closely packed that they form a series of 3D walls of a well-defined structure generally observed for porous materials. The majority of pores have walls with wall thicknesses on the order of one micrometer, though thinner (<1 µm) and thicker walls are also observed. One can see isolated pores (see Figure 6c-right) with micrometer or submicrometer dimensions, while connected pores form crevice-like structures.

The influence of chitosan can be well observed in the two-component surface of nanofiber films, as evidenced by the SEM micrographs. Thus, in Figure 6d, one can see the SEM images of Ch6AA90(50%)FG6H_2_O(50%) nanofiber film surface. On a chitosan-based surface, one can see an isolated formation, in parts forming clusters of almost spherical orbs. These have diameters on the order of one micrometer or less and play the role of origin/end points for several thin fibers, with an estimated diameter under 100 nm. To each spherical orb, a large number of such thin fibers are connected, which, where present, form a dense network of well-oriented fibers. Thus, one can remark that the thin fibers present a tendency to join two orbs together rather than interconnect among themselves. Nevertheless, Figure 6d-right also presents some localized sub-networks of even thinner fibers that connect the main network.

The Ch6AA90(90%)PVA6AA90(10%) nanofiber film presents the most structured fibrillary network. Thus, in Figure 6e-left, one can see a series of 3D quasi-spherical or elliptically shaped bulbs that are connected via thick fibers. The thickness of these fibers can be well estimated from Figure 6e-right with a sub-micrometer thickness of 200–300 nm and lengths ranging from ~1 µm to several tens of micrometers. Along this main fiber may or may not be present secondary bulbs (with diameters much smaller than those of the main bulbs). In general, these secondary bulbs may act as junction points between multiple main fibers. However, simple chemical cross-links may exist between the main fibers without bulbs. One can consider that the main fibers form a so-called mesh network. Secondary fibers with diameters ranging from half to ~one tenth of the diameter of the primary fibers form a local secondary network. These are characterized by a length of submicrometers to several micrometers and usually are connected to the primary network directly, though they may also be connected through small bulbs. Several tertiary fibers can form another local network and join the secondary fibers via direct links. A tertiary fiber can join two secondary fibers without other contacts, although some tertiary fibers are cross-linked at only a few points. At this magnification, quaternary fibers are difficult to observe.

Chitosan is an excellent film-forming material with good mechanical properties that allows for easy manipulation of produced films and, at the nano- to micrometer scale, leads to a smoother film surface. This effect was most obvious in the case of Ch6AA90(50%)PEG6AA90(50%) electrospun film. The SEM images measured for this sample are presented in Figure 6f. The nanofibers are barely observable as relatively short, thin lines on top of the remains of walls. Almost all pores are significantly filled, and thus this two-component PEG/chitosan film presents only a series of shallow open pores (see Figure 6f-right). Quasi-spherical formations are also distributed across the film surface.

#### 3.3.2. Structural Characterization by FT-IR Spectroscopy

**Figure 7:**
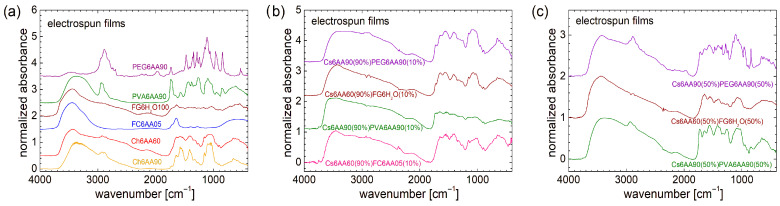
FT-IR spectra measured for film electrospun from (**a**) pure solution of acetic acid (90% or 60% and distillated water) and 6% *w*/*v* chitosan, fish gelatin, PVA, and PEG; (**b**) 90% solution 1 of 6% *w*/*v* chitosan (AA 90% or 60%) and 10% of solution 2 of 6% *w*/*v* marine fish collagen (AA 5%), fish gelatin (100% distillated water), PVA (AA 90%), and PEG (AA 90%); (**c**) 50% solution 1 of 6% *w*/*v* chitosan (AA 90% or 60%) and 50% of solution 2 of 6% *w*/*v* marine fish collagen (AA 5%), fish gelatin (100% distillated water), PVA (AA 90%), and PEG (AA 90%).

From a molecular–structural point of view, as can be observed from FT-IR spectral analysis, one can say that PEG (in a PEG-based one-component film, i.e., PEG6AA90) presents the most complex and well-defined structure (see Figure 7a). This FT-IR spectrum is more enhanced compared with that measured for the raw PEG (see Figure 2a), presenting more resolved peaks. Conversely, in the case of PVA, the FT-IR spectral features are less pronounced (compared with those observed for raw PVA) in the sense that the lines are broadened, and some new lines appear, as is the case of those located at ~1736 cm^−1^ or the wide water peak centered at ~3380 cm^−1^. A certain structure can also be observed in the FT-IR spectrum measured for the Ch6AA90 film, while a reduced amount of acetic acid leads to a broadening of the lines of the obtained Ch6AA60 film. Almost no distinct features can be observed in the FT-IR spectra measured for marine-fish-collagen- and fish-gelatin-based one-component bio-films (e.g., FC6AA05 and FG6H_2_O100).

As was the case for the solutions, the FT-IR spectra recorded for unbalanced (90:10) two-component nonwoven films present similar features imposed by the large quantity of chitosan (see Figure 7b). In the range of low wavenumbers (<1850 cm^−1^), the FT-IR spectra measured for Cs6AA90(90%)PEG6AA90(10%) and Cs6AA60(90%)FG6H_2_O(10%) films present almost the same features of quasi-resolved broad peaks imposed by the high content of Cs6AA60(90%). Then, one can add the marine-fish-collagen-based film (Cs6AA60(90%)FC6AA05(10%)), for which the corresponding FT-IR spectrum is slightly more structured, in the sense that one can observe some narrow peaks with low amplitude on top of the broad peaks. The FT-IR spectrum measured for Cs6AA60(90%)FG6H_2_O(10%) nanofiber film is less structured, with almost no structure in this range of wavenumbers. In fact, this feature (almost no structure) can be found in the FT-IR spectra measured for all unbalanced two-component films. One can remark some differences in the range of 2800 cm^−1^ to 3000 cm^−1^, where, for example, a faint trace of the specific peak of PEG is still observed.

An increased content of PEG- and PVA-based solutions enhanced the features of FT-IR spectra measured for the corresponding balanced two-component films, i.e., Cs6AA90(50%)PEG6AA90(50%) and Cs6AA90(50%)PVA6AA90(50%), as can be observed from Figure 7c. In the range of low wavenumbers (<1850 cm^−1^), the FT-IR spectrum measured for the PEG-based balanced two-component films closely resembles that of those measured for the one-component film (see Figure 7a), where a series of narrow and well-resolved peaks are observed. Moreover, in the range of 1490 cm^−1^ to 1830 cm^−1^, the FT-IR spectrum measured for Cs6AA90(50%)PEG6AA90(50%) *imports* some features from the FT-IR spectrum of the Cs6AA90 one-component film. In fact, with the exception of some specific features that can be observed in the FT-IR spectra of PEG6AA90 and PVA6AA90 films in the range of 2800 cm^−1^ to 3000 cm^−1^, the above-mentioned import can be observed for an extended range of wavenumbers, i.e., from 1490 cm^−1^ up to 4000 cm^−1^. And since the wavenumber can reflect the amount of energy absorbed by the sample, e.g., the vibrational frequency of chemical bond vibrations, then one can say that (i) the overall backbone structure of the PEG- and PVA-based unbalanced two-component films, which vibrate at low frequencies (reflected in the low wavenumber), is influenced by these PEG and PVA polymers; and (ii) small structures and functional groups that vibrate at higher energy (reflected in the high wavenumber) are influenced mainly by chitosan. Conversely, at the molecular level, the structure of the two-component nanofiber films based on fish gelatin is less sensitive to FG6H_2_O, as can be deduced from the small changes that are observed in the measured FT-IR spectra (see brown spectra in Figure 7b,c). One may claim that it is due to the fish gelatin, but taking into consideration the above discussion, the presence of water (or the absence of acetic acid in the second component) may also play a significant role.

#### 3.3.3. Polymer Network Dynamics Evaluated by Low-Field ^1^H NMR Relaxometry

**Figure 8:**
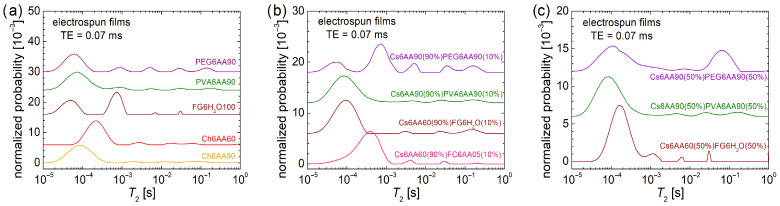
Low-field ^1^H NMR *T*_2_- distributions measured for the films electrospun from (**a**) pure solution of acetic acid (90% or 60% and distillated water) and 6% *w*/*v* chitosan, fish gelatin, PVA, and PEG; (**b**) 90% solution 1 of 6% *w*/*v* chitosan (AA 90% or 60%) and 10% of solution 2 of 6% *w*/*v* marine fish collagen (AA 5%), fish gelatin (100% distillated water), PVA (AA 90%), and PEG (AA 90%); (**c**) 50% solution 1 of 6% *w*/*v* chitosan (AA 90% or 60%) and 50% of solution 2 of 6% *w*/*v* marine fish collagen (AA 5%), fish gelatin (100% distillated water), PVA (AA 90%), and PEG (AA 90%).

Once the nanofibers (with evident or less evident structure, as can be seen from SEM images) are produced by electrospinning, the most mobile components (acetic acid, water) largely—but not completely—evaporate. The experimental procedure assumes, prior to use, a rest time when the majority of the volatile components continue to evaporate. As a consequence, the produced nonwoven films, which are in a solid form, present again a rigid consistency. This property can be well characterized by low-field ^1^H NMR relaxometry. In this sense, the *T*_2_-distribution measured for one-component and two-component films is presented in Figure 8.

As expected, the normalized *T*_2_-distribution measured for films resembles more those measured for the raw materials (see Figure 2b and corresponding figures from ref. [4]). For the one-component films, the main peaks (largest integral area) are located in the position described by low *T*_2_-values, indicating that the majority of ^1^H is in components with strongly reduced mobility (see Figure 8a). Compared to the rest of the four distributions, which present a single main component, that of FG6H_2_O100 presents two main peaks, well resolved, associated with rigid components with different restricted mobility. The assumption that being the single sample that does not contain acetic acid, but water, which is not so volatile as AA, and therefore for which the evaporation process is not so fast, may be a hypothesis that can be considered with a certain degree of probability. From the rigidity point of view, as inferred from the position of the main peak in the *T*_2_-distributions, one can say that PEG6AA90 films with a *T*_2_ ≈ 61.7 µs is the most rigid, followed closely by PVA6AA90 film (*T*_2_ ≈ 71.5 µs), then by Ch6AA90 film with a *T*_2_ ≈ 87.0 µs, and the less rigid film is Ch6AA60 film with a *T*_2_ ≈ 215.4 µs. However, one can report that the PEG6AA90 film is just apparently rigid, when in fact, from a mechanical point of view, the samples crumbled easily. Conversely, Ch6AA90 and, especially, Ch6AA60 films are more flexible. This was one of the reasons why Ch6AA90 was chosen to be one of the two components for the two-component films. One could also choose PVA6AA90, which also presents good mechanical properties at manipulation, but it was our decision to produce some nanofiber films using bio-polymers as much as possible. The other properties of chitosan described in the introduction also play an important role. With the two main components, located at *T*_2_ ≈ 48.3 µs and *T*_2_ ≈ 723.9 µs, the biofilm can also be well manipulated and represents a good alternative to chitosan. The solution FC6AA05 could produce films, but these present a large adhesion to the support that could not be unglued and therefore are not characterized further. The mechanical (and not only) properties are not given only by the most rigid component, and the low peaks characterizing components containing ^1^H, with different mobility. In this sense, one can observe that the electrospun PEG6AA90 film presents the largest number (and amount) of such mobile components, as can be seen from the corresponding *T*_2_-distribution. One can also observe that the concentration of AA plays an important role in the *T*_2_-distribution measured for the electrospun chitosan-based samples. Less acid translates into more water, leading to increased mobility. This was exactly the observation pointed out previously for the FG6H_2_O100 nanofiber film, which, according to the peaks located at high *T*_2_-values, presents the most structured (homogeneous), but fewer in quantity, components characterized by increased mobility.

Among all unbalanced (90:10) two-component films (see Figure 8b), that one which contains PVA (i.e., Cs6AA90(90%)PVA6AA90(10%)) presents the corresponding *T*_2_-distribution most similarly to that recorded for the one-component film Ch6AA90 (see the bottom distribution in Figure 6a). One can observe that in the case of a two-component film, the *T*_2_-distribution presents only slightly elevated peaks corresponding to highly mobile components. Then, one can conclude that PVA (among all used materials in this study) has the smallest influence on the dynamics of the produced nanofiber films. Conversely, PEG has a huge influence on the distribution of components by their mobility, as can be observed from the *T*_2_-distribution measured for the electrospun Cs6AA90(90%)PEG6AA90(10%) film. One can observe a large contribution of components with increased mobility, but which are still maintaining their identity (the peaks are well resolved). The large difference is obtained in the range of low *T*_2_-values. Here, one can observe that the main peak is located at higher *T*_2_-values (*T*_2_ ≈ 715.5 µs) than those observed for each of those two components, separately. The measured *T*_2_-distribution for the electrospun Cs6AA90(90%)PEG6AA90(10%) film is not a superposition of the *T*_2_-distributions of one-component films, indicating that in solution and during the electrospinning process, complex interactions take place between the constituting components. As a PVA, fish gelatin in a solution of FG6H_2_O and 10% in the two-component nanofiber film changes, only to a small extent, the *T*_2_-distribution compared with the distribution measured for Cs6AA90 (see Figure 8a). Here, one can not only find the main peak located at *T*_2_ ≈ 94.8 µs but also an elevated peak describing the most mobile components. In a small proportion and in combination with Cs6AA90, the marine fish collagen can form a usable nanofiber film. Among all two-component films, this film (labeled Cs6AA60(90%)FC6AA05(10%)) presents the most rigid component at the most elevated *T*_2_-values (*T*_2_ ≈ 387.6 µs), which may explain the poor mechanical properties.

At a high content, e.g., balanced (50:50) two-component samples, Cs6AA60(50%)FC6AA05(50%), were not able to form usable nanofiber films and were therefore not characterized. Thus, only three balanced two-component electrospun films are reported and characterized (see Figure 8c). One can observe that even this elevated content of PVA did not significantly change the *T*_2_-distributions measured for the balanced two-component nanofiber film (Cs6AA90(50%)PVA6AA90(50%)). Compared to the unbalanced two-component film, one can observe just a small increase in the integral area of peaks associated with the mobile components. This is an indication that PVA interacts well with the acetic acid at the creation of PVA6AA90 solution, and the resulting two-component electrospun film mainly presents the characteristics originating from the chitosan-based solution (Cs6AA60). As in the case of the unbalanced two-component film, one can observe an increase in the PEG polymer in PEG6AA90 solution, also on the balanced two-component film Cs6AA90(50%)PEG6AA90(50%). In this sense, in the measured *T*_2_-distribution, one can observe a large peak (broad and with a large integral area) corresponding to the most mobile and semi-mobile components. At an elevated content of PEG, these two peaks form a single peak, indicating an increase in heterogeneity. The same increase in heterogeneity is observed also for the less mobile components, which also present only one broad peak. The specific features of *T*_2_-distribution observed for the one-component film (see FG6H_2_O in Figure 8a) start to be observed also in the balanced two-component nanofiber film Cs6AA60(50%)FG6H_2_O(50%) (see Figure 8c). The contribution of mobile components is slightly elevated, indicating that this component is not primarily affected by mixing and electrospinning. Indeed, the major changes take place at the level of the most rigid component with the apparition of only one (major) peak located at *T*_2_ ≈ 164.7 µs. This is a value larger than those measured for the major peak of the one-component Cs6AA90 and between the two values measured for the one-component FG6H_2_O. From an NMR relaxometry point of view, among all bio- or organic polymers, one can say that, in combination with the chitosan solution, a fish gelatin solution can better mediate the properties of the produced nanofiber film. However, this result is not surprising since fish gelatin alone can also form bio-nanofiber films with good mechanical properties.

#### 3.3.4. Molecular Exchange Evaluated from ^1^H NMR *T*_2_-*T*_2_ EXSY Maps

**Figure 9:**
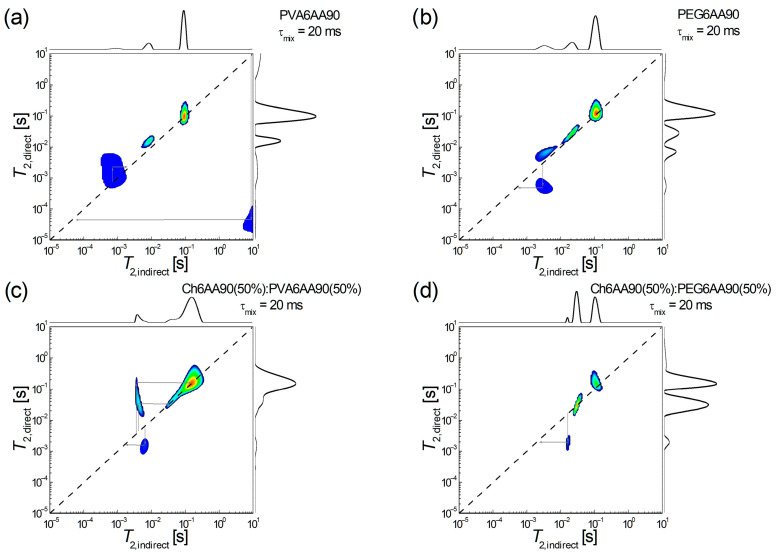
^1^H NMR *T*_2_-*T*_2_ EXSY spectroscopy measured for films electrospun from solutions of one component: (**a**) PVA 6 *w*/*v* in 90% acetic acid, (**b**) PEG 6 *w*/*v* in 90% acetic acid, and balanced (50:50) two-component solutions based on (**c**) chitosan 6 *w*/*v* in 90% acetic acid (sol-1) and PVA 6 *w*/*v* in 90% acetic acid (sol-2) and (**d**) chitosan 6 *w*/*v* in 90% acetic acid (sol-1) and PEG 6 *w*/*v* in 90% acetic acid (sol-2).

The low-field ^1^H NMR *T*_2_-*T*_2_ EXSY has recently been proven to be able to characterize the molecular exchange in the complex network as that of the recently produced electrospun films [4]. Shortly, the interpretation is in terms of diagonal peaks associated with ^1^H pools (or reservoirs) and extra-diagonal peaks that indicate the presence of an exchange between hydrogen pools. A series of 4 ^1^H NMR *T*_2_-*T*_2_ EXSY maps are presented in Figure 9, two for one-component and two for two-component films produced by electrospinning. Thus, in Figure 9a, the two-dimensional ^1^H NMR *T*_2_-*T*_2_ exchange map measured for PVA6AA90 nanofiber film is presented. One can observe a series of three *diagonal* peaks and one extra-diagonal peak. From those three peaks located on the main diagonal, (i) the one which appears at *T*_2,direct_ = *T*_2,indirect_ ≈ 91.2 ms is on-diagonal; (ii) the one which appears at *T*_2,direct_ ≈ *T*_2,indirect_ ≈ 10 ms is just a little bit off-diagonal (which may attributed to the limited number of radiofrequency pulses in the 2D pulse sequence, thus a limited acquisition); (iii) the one which is located at *T*_2,direct_ ≈ *T*_2,indirect_ ≈ 1 ms may be considered a superposition of a diagonal peak and an exchange between ^1^H pools of semi-rigid components with similar mobility (*T*_2,indirect_ ≈ 0.71 ms → *T*_2,direct_ ≈ 2.18 ms) into a process that assumes a slight increase in mobility. Another exchange process can clearly be seen between components (^1^H pools) with extreme mobility. Thus, one can observe some highly mobile components, probably some unevaporated solvent fractions, which may contain acetic acid, will form/contribute to some extreme rigid components (*T*_2,indirect_ ≈ 8.7 s → *T*_2,direct_ ≈ 44.6 µs). This exchange map demonstrates that the PVA6AA90 nanofiber film is not completely dry even after several days after preparation.

An exchange process, but between components with relatively similar mobility, is also observed for the PEG6AA90 electrospun film, as can be seen in Figure 9b. Also, in this case, a stiffening process is observed (*T*_2,indirect_ ≈ 2.94 ms → *T*_2,direct_ ≈ 0.49 ms) from a medium mobile component to a semi-rigid component. Since no other exchange peaks are observed, then one can conclude that, at the molecular level, the PEG6AA90 film is completely dry. As in the case of PVA6AA90, we see a series of three diagonal peaks, one discussed (the source of exchange) at two stabiles with increased mobility.

Both two-component electrospun films are completely dry, since the corresponding PVA- and PEG-based 2D ^1^H NMR *T*_2_-*T*_2_ EXSY maps do not show any extra-diagonal peaks originating from high *T*_2_-values (see Figure 9c,d). Nevertheless, one can see that PVA leads to more active nanofiber polymeric fibers (Figure 9c) presenting both exchanges: (i) a two-step increase in mobility; first one can see an exchange from medium mobile components to the most mobile components (*T*_2,indirect_ ≈ 3.59 ms → *T*_2,direct_ ≈ 164.5 ms) and then from almost the same medium mobile components to semi-mobile ^1^H pools (*T*_2,indirect_ ≈ 4.11 ms → *T*_2,direct_ ≈ 33.7 ms); (ii) a stiffening process originating from medium mobile components to slightly less medium-mobile components (*T*_2,indirect_ ≈ 6.45 ms → *T*_2,direct_ ≈ 1.55 ms). As in the case of PEG based one-component electrospun film (see Figure 9b) the balanced two-component electrospun film Ch6AA90(50%):PEG6AA90(50%) presents a single exchange process, as can be deduced from the existence of a single extra-diagonal exchange peak observed in Figure 9d. This leads to a stiffening of the film as observed from a ^1^H pool with an increased medium mobility (*T*_2,indirect_ ≈ 15.8 ms) to a ^1^H pool with a reduced medium mobility (*T*_2,direct_ ≈ 1.95 ms). Another two pools associated with ^1^H pools (components) with an increased mobility are also observed.

Solid-like to liquid-like dynamic components evaluated from ^1^H NMR *T*_1_-*T*_2_ COSY spectroscopy

**Figure 10:**
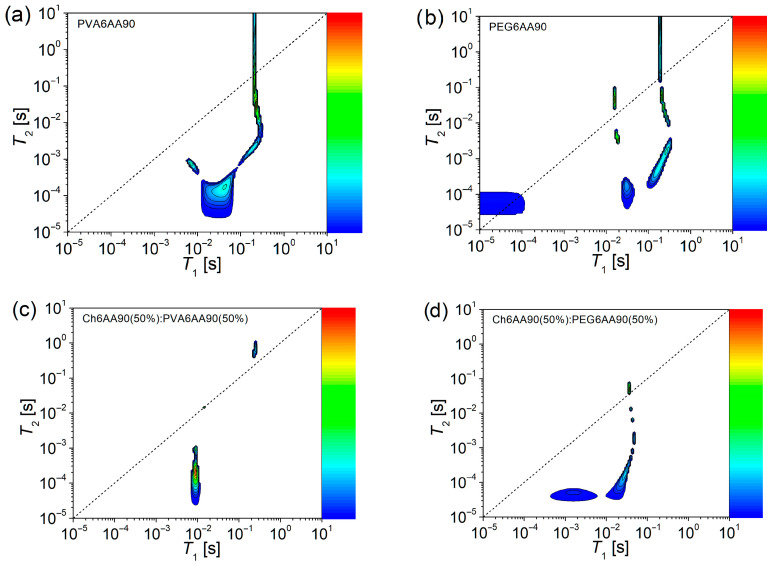
The 2D ^1^H NMR *T*_1_-*T*_2_ COSY spectroscopy measured for films electrospun from solutions of one component (**a**) PVA 6 *w*/*v* in 90 % acetic acid, and (**b**) PEG 6 *w*/*v* in 90 % acetic acid, and balanced (50:50) two component solutions based on (**c**) chitosan 6 *w*/*v* in 90% acetic acid (sol-1) and PVA 6 *w*/*v* in 90% acetic acid (sol-2) and (**d**) chitosan 6 *w*/*v* in 90% acetic acid (sol-1) and PEG 6 *w*/*v* in 90% acetic acid (sol-2).

Usually, two-dimensional ^1^H NMR *T*_1_-*T*_2_ COSY can reveal more complex dynamic components than the 2D ^1^H NMR *T*_2_-*T*_2_ exchange maps. For a comprehensive understanding, the corresponding *T*_1_-*T*_2_ correlation maps are presented in Figure 10, for the same electrospun films discussed above. As in the case of *T*_2_-*T*_2_ EXSY maps (Figure 9), one can observe a main diagonal represented by a short-dashed line in Figure 10. This line topologically indicates the positions in maps where the measured spin-lattice relaxation time and the spin-spin relaxation time are equal (*T*_1_ = *T*_2_). If some peaks appear close to this main diagonal, then they can be associated with components for which the molecular motion is strongly averaged out due to the fact that the inverse of the correlation time is much higher compared to the Larmor frequency (1/τ_c_ ≫ ω_0_). Thus, the peaks that appear in the proximity of the main diagonal can be associated with the liquid-like components [4,5,6,7]. The peaks located below the main diagonal indicate that the *T*_2_ relaxation process is faster than the *T*_1_ relaxation process. This is the case of fluids and viscous fluids, where molecules can form aggregates. Here, the molecular motion is no longer averaged out and *T*_2_ becomes much smaller than *T*_1_ [6]. For quasi-solids and solids (from elastomers to polymers), the ratio *T*_2_/*T*_1_ < 1 indicates a solid-like component. In the case of electrospun films, usually the solid-like components can be part of polymer chains with restricted mobility subjected to various restrictions, such as interactions with neighboring polymer chains, and chemical and/or physical crosslinks. Otherwise, the liquid-like components can be formed from dangling chains, end-chains, or unevaporated solvent.

The 2D ^1^H NMR *T*_1_-*T*_2_ COSY measured for the PVA6AA90 nano-fiber film presents a series of peaks, indicating the presence of both solid-like and liquid-like components and more (see Figure 10a). First, one can see an extended peak centered at *T*_1_ ≅ 42.7 ms and *T*_2_ ≅ 0.17 ms, which extends towards smaller *T*_1_- and *T*_2_-values, indicating a solid-like component that presents a certain degree of heterogeneity. Starting from here, a component *extends perpendicular* to the main diagonal (centered at *T*_1_ ≅ 7.1 ms and *T*_2_ ≅ 0.74 ms), indicating a transition of character from solid-like to quasi-liquid-like. Another component extends parallel to the main diagonal, indicating the preservation of the solid-like component, but as *T*_2_-values increase, the mobility of this component also increases. This increase is maintained only up to a point, where the *T*_2_-values continue to increase but not the *T*_1_-values. Therefore, one can conclude that, with the increase in mobility, one can also observe the change in the character from solid-like to liquid-like and beyond as the *T*_2_-values become larger than the *T*_1_-values.

The above-described behavior is also observed for the one-component PEG6AA90 electrospun film, but with two distinct features (see Figure 10b). Thus, the component that can be characterized as the most solid-like is centered at *T*_1_ ≅ 30.4 ms and *T*_2_ ≅ 0.17 ms, but extends only along the *T*_2_-direction, indicating a reduced heterogeneity compared to the PVA6AA90 electrospun film. A more extended and better-defined component, compared to the case of PVA6AA90 sample extends *quasi-parallel* to the main diagonal. In fact, with the increase of *T*_2_-values, the peaks approached to the main diagonal, indicating that the increase in mobility leads to a slight change in the character becoming less solid-like. As was mentioned above, the limit is observed, together with the change in the character from solid-like to liquid-like at almost the same *T*_1_- and *T*_2_-values as in the case of PVA6AA90 film. But this change is not unique since one can observe another two peaks located closely to the main diagonal (one below and one above), which may represent some liquid-like components with reduced mobility. Surprisingly, one can observe another peak presenting some liquid-like behavior but characterized by a reduced mobility, possibly confined within a restricted geometry (*T*_1_ ≅ *T*_2_ ≅ 50 µs).

The 2D ^1^H NMR *T*_1_-*T*_2_ COSY measured for the two-component nanofiber films show a higher degree of structural organization (homogeneity) especially in the case of the Ch6AA90(50%)/PVA6AA90(50%) sample (see Figure 10c). Here, one can see only three peaks, with a small surface area, thus more homogeneous. Among them, the solid-like component presenting an associated peak in the 2D *T*_1_-*T*_2_ COSY map centered at *T*_1_ ≅ 9.27 ms and *T*_2_ ≅ 0.21 ms but extending along the *T*_2_ axis. Another one, exactly on the main-diagonal describing a pure liquid-like behavior (*T*_1_ ≅ *T*_2_ ≅ 14.8 µs), presents the smallest surface in the 2D *T*_1_-*T*_2_ COSY map. And finally, a third peak appears slightly above the main diagonal centered at *T*_1_ ≅ 251 ms and *T*_2_ ≅ 697 ms, probably associated with the most mobile components. The 2D ^1^H NMR *T*_1_-*T*_2_ COSY map measured for Ch6AA90(50%)/PEG6AA90(50%) electrospun film (see Figure 10d) is more complex than the Ch6AA90(50%)/PVA6AA90(50%) film. At the same time, it closely resembles the map of the one-component PEG6AA90 electrospun film (see Figure 10b), but shifted and simplified. Nevertheless, the influence of PEG remains visible, and one can observe a series of components, such as those characterized as solid-like and centered at *T*_1_ ≅ 28.5 ms and *T*_2_ ≅ 0.17 ms, from which an increase in mobility (of other components with increased *T*_2_-values) also leads to a change in the character from solid-like to liquid-like, for which the most mobile component presents a peak that touches, in the 2D *T*_1_-*T*_2_ COSY map, the main diagonal. The effect of chitosan is to *accelerate* this transition (e.g., at lower *T*_1_-values). At the same time, the chitosan-based component changed the *T*_2_/*T*_1_ ratio (expressed as the change in position in the *T*_1_-*T*_2_ COSY map) of the most rigid component as was observed for one-component PEG6AA90 (Figure 10b) compared to that observed for the two-component (Figure 10d). This change can be interpreted as being mediated by long-range interactions, since the *T*_2_-values (responsible to short range interactions) are found in the same range, but the *T*_1_-values increase with almost two orders of magnitude.

#### 3.3.5. Application and Machine Learning for the Characterization of the Orientation of Bio-Nanofibers

**Figure 11:**
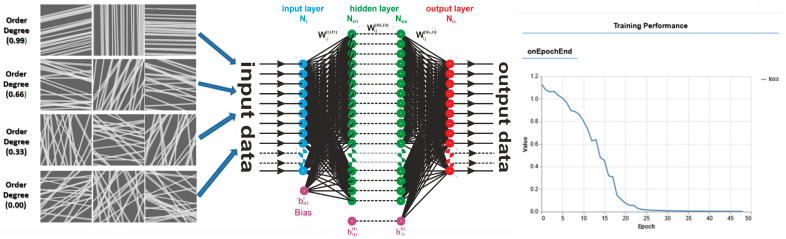
Principle of machine learning algorithm used to train and then to predict the orientation of nanofibers from SEM images.

The mechanical properties of fibrillary materials are greatly influenced by the orientation of the constituting fibrils. For oriented fibrillary materials, a certain degree of anisotropy between the fiber axis and the perpendicular direction can be expected. For randomly oriented fibers, the influence of anisotropy on the properties is expected to be reduced. Therefore, the knowledge of the orientation degree (or order degree) plays an important role in the material’s engineering. We propose to take advantage of the measured SEM images and to estimate the order degree from these by implementing a machine learning algorithm based on a trained artificial neural network in this sense. The schematic representation of this numerical set-up is presented in Figure 11. At the center is the ANN architecture, consisting of an input layer, several hidden layers, and an output layer. As input data, we provided a large set (800 from a total of 1000) of synthetic images labeled with their corresponding order degree (see the left side of Figure 11). To generate these images, a simple program was written in Processing language.

An order degree of 1 represents a perfectly ordered fiber network, while a zero-order degree represents a totally disordered network. The ANN was trained for several epochs, not for classification, as is typical in most image analysis procedures, but for regression. This means that the ANN is able to predict the order degree by providing a normalized value (between 0 and 1), which was the primary reason for associating the order degree to this range. The training efficiency is represented by the loss parameter, and the training performance is displayed as a graph in the right panel of Figure 11. As one can see, the ANN was efficiently trained in fewer than 50 epochs.

**Figure 12:**
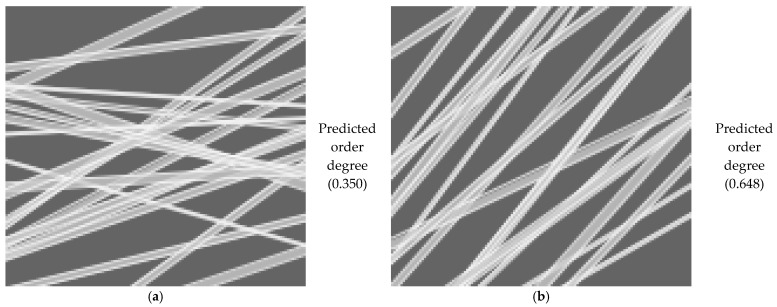

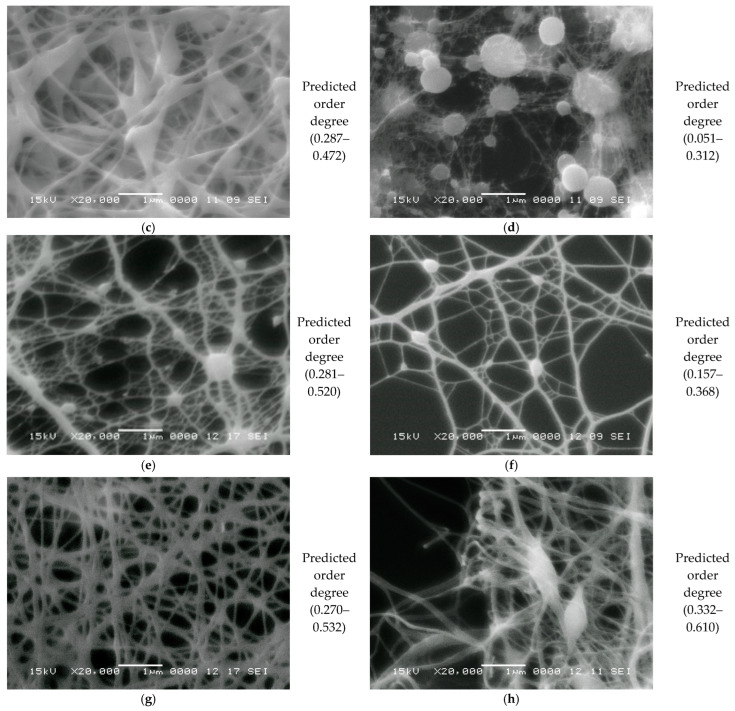
The order degree of electrospun nanofibers predicted by ANN from simulated fibers with (**a**) 0.33 and (**b**) 0.66 order degrees and SEM images measured with a magnification of 20000 for (**c**) FG6H_2_O; (**d**) Ch6AA90(50%)FG6H_2_O(50%); (**e**,**f**) Ch6AA90(90%)PVA6AA90(10%); and (**g**,**h**) PVA6AA90.

The accuracy of training was tested on other images not included in the training set of synthetic images (200 out of a total of 1000). The results for two images are presented in the first row of Figure 12. One can observe that the predicted order degree for the image (left image from first row of Figure 12) synthesized with an order degree of 0.33 was 0.350. To this prediction, one can associate a relative error of ~6.07%. The predicted order degree for the image simulated with an order degree of 0.66 was 0.648. The associated relative error was ~1.82%. Therefore, it is safe to conclude that the relative error in predicting the order degree (degree of fiber orientation) is less than 10%.

The analysis and prediction procedure was applied on some of the measured SEM images. It was first observed that the predicted order is not a single value but is better described as a range. This is a reasonable approach for nanofibers with so many characteristics other than linear fibers (see the previous discussion related to the characterization of SEM images). Thus, one can observe that the one-component FG6H_2_O fish-gelatin-based nanofiber film presents an order degree in the range of 0.287–0.472. This means that the fibers are mostly disordered. The most disordered nanofibers were found for the two-component Ch6AA90(50%)FG6H_2_O(50%) also based on fish gelatin. The predicted order degree was in the range of 0.051–0.312, placing this network as being composed of nearly totally disordered nanofibers. The density of the nanofiber network can play also an important role in the order-degree analysis, as can be seen from the set of two images presented in Figure 12e,g (third row) for the two-component Ch6AA90(90%)PVA6AA90(10%) nanofiber film. As expected, a denser network can be characterized by a more heterogeneous fiber orientation with a degree order in the range of 0.281–0.520. Here, one can see that some parts of nanofiber network exceed the order–disorder threshold value of 0.5. A less dense network presents a narrower range of order degree (0.157–0.368) and, overall, can be characterized as more disordered than the denser network. The last two predictions (bottom row in Figure 12) made for the PVA-based films, the two-component Ch6AA90(90%)PVA6AA90(10%), and one-component PVA6AA90 nanofiber films show that the degree of disorder may increase with the number of components. Thus, the one-component PVA6AA90 nanofiber network may exhibit sections with a higher degree of orientation, as can be deduced from the highest value of degree order in the range of 0.332–0.610 predicted for this sample. One can see that the predicted range for the one-component PVA6AA90(10%) nanofiber network (0.270–0.532) is very close to that predicted for the two-component Ch6AA90(90%)PVA6AA90(10%) nanofiber network (i.e., 0.281–0.520), even if the SEM images clearly reveal distinct network morphologies.

## 4. Discussions and Perspectives

Nanofibers produced using electrospinning machines from natural and synthetic polymers represent a promising approach with significant applications in various fields, such as medicine and materials engineering. Collagen alone, even in its non-denatured form, rarely forms stable solid nanofiber films that may be used in practical applications. However, it has been shown that two-component, collagen-containing nanofiber films may be produced by electrospinning when the collagen-based solution has a concentration below 50%. As a second component, chitosan is an excellent biomaterial, strengthening the solid nanofiber films and making the final product usable in diverse practical applications. Nevertheless, one should expect that, when the chitosan content is high (≥60% in solution, as in the cases presented in this study), it tends to fill the inter-fiber spaces of the network formed by the second component, and distinct nanofibers are no longer formed. The usage of fish gelatin, alone or in tandem with other (bio)materials, for electrospinning presents a series of advantages. Fish gelatin can be dissolved in water (instead of acids such as acetic acid) and can form as mono- or bi-component nanofibers forming highly disordered networks. PVA is an organic polymer that can successfully replace chitosan to form solid nanofibers films with good mechanical properties, which can be easily manipulated and thus used in practical applications. Alone or in combinations with chitosan can form long nanofibers, which are arranged into a complex network described as hierarchically structured and which can exceed the disorder-to-order threshold of 0.5. PEG alone can form solid films, but these are fragile and cannot be used in practical applications. Nevertheless, in combination with another material, such as chitosan (presented here) or PVA (not discussed in this study), it can form two-component usable solid nanofiber films. At the micro- or nanometric level, distinct fibers are not observed, while the surfaces present an *organic*-like cohesive morphology. From a dynamic point of view, this study shows that all samples exhibit a large number of dynamic components, and the stiffening is a continuous process.

To further capitalize on the findings presented in this article, we will briefly discuss some typical biomedical applications of electrospun nonwoven materials, such as tissue engineering, wound healing, drug delivery systems, and bio-membranes for filtrations. Thus, in tissue engineering, the action mechanism is based on a product that is characterized by a micro-architecture that can mimic the natural extracellular matrix and may promote the cell adhesion, migration, and differentiation, while finally, the produced scaffolds biodegrade into new tissue forms. The typical materials might include combinations of PCL(Poly(ε-caprolactone))/gelatin, PLGA(Poly(lactic-co-glycolic acid))/collagen, and chitosan/silk fibroin. Additionally, the nanofiber nonwoven materials have to be characterized by some particular properties, such as (i) high interconnected porosity, which may enable cell infiltration and nutrient diffusion—which can be evaluated by SEM (classical) but more dynamical by 2D ^1^H NMR *T*_2_-*T*_2_ exchange spectroscopy; (ii) uniform fiber alignment and morphology for controlling cell orientation—evaluated by AI-enhanced SEM image analysis; (iii) medium crystallinity to provide mechanical stability and gradual degradation—evaluated by XRD; (iv) stable chemical bonding between biopolymers (amide, hydroxyl peaks)—evaluated by FT-IR; (v) moderate water mobility and controlled hydrolysis during degradation—evaluated by low-field 1D and 2D ^1^H NMR *T*_2_-*T*_2_ EXSY and *T*_1_-*T*_2_ COSY.

Another important biomedical application of electrospun nonwoven materials is wound healing, where the nanofiber materials must form a breathable yet moist barrier that accelerates tissue regeneration, releases antimicrobial agents, and protects against bacterial infection. Typical nanofiber materials are based on PVA/chitosan, collagen/PEG, gelatin/AgNPs (silver nanoparticles), or PCL/aloe vera. The materials have to be characterized by some particular properties, such as (i) high surface area and micro-porosity for rapid exudate absorption—evaluated by SEM (classical) and dynamically by 2D ^1^H NMR *T*_2_-*T*_2_ exchange spectroscopy; (ii) strong hydrogen bonding and polymer compatibility (amide, OH bands)—evaluated by FT-IR; (iii) medium hydration capacity and moisture retention to maintain a moist healing environment—evaluated by low-field 1D and 2D ^1^H NMR *T*_2_-*T*_2_ EXSY (as discussed in this study) and also by low-field 1D ^1^H NMR diffusiometry (not discussed here); (iv) high structural integrity even under wet conditions—evaluated by low-field 1D and 2D ^1^H NMR *T*_2_-*T*_2_ EXSY, FT-IR, and XRD.

As a drug delivery system (DDS), the electrospun nanofiber material should enable programmable and localized drug release through diffusion and polymer degradation, with release kinetics tailored by fiber design. Some typical examples of electrospun materials may include coaxial PLGA/PCL and PVA/PEG nanofibers loaded with drugs. For that, the electrospun nanofibers may exhibit some key properties, such as (i) precisely defined core–shell morphology ensuring encapsulation efficiency—which classically may be evaluated by SEM but modern may be the subject of by low-field 1D and 2D ^1^H NMR *T*_2_-*T*_2_ EXSY and even by AI segmentation; (ii) controlled crystallinity reduction after drug loading—evaluated by XRD; (iii) distinct drug–polymer interaction signatures confirming molecular binding and compatibility—evaluated by both FT-IR spectroscopy and by high-field solid-state 1D or 2D (COSY, NOESY) NMR spectroscopy or by low-field 1D and 2D ^1^H NMR *T*_2_-*T*_2_ EXSY relaxometry; (iv) low-diffusional resistance and tunable porosity for sustained release—evaluated by low-field 1D and 2D ^1^H NMR *T*_2_-*T*_2_ EXSY and *T*_1_-*T*_2_ COSY relaxometry complemented by low-field ^1^H NMR diffusiometry.

Finally, for bio-membranes used in filtration, the nanofiber nonwoven materials must physically trap or adsorb microorganisms and biomolecules through size exclusion and/or surface affinity and must be tuned for biocompatible or selective separation. Some typical examples include PCL, PAN (Poly(acrylonitrile)), and PVDF (Poly(vinylidene fluoride)) nanofiber filters. In order to accomplish the filtration function, the electrospun nanofibers should present important properties, such as (i) high nanoporous density enabling bacterial and viral retention—which classically is evaluated by SEM, but also the low-field 1D ^1^H NMR relaxometry is currently used for the determination of pore size distribution; (ii) moderate structural order and crystallinity ensuring durability—which may be evaluated by XRD, by our proposed SEM-ANN methods, or also by various methods of low-field NMR, such as the presented relaxometry (*T*_2_-distributions) or even enhanced by those based on spin-diffusion; (iii) surface functionalization with hydrophilic or charged groups for selective capture—which may be well evaluated from FT-IR and solid-state high-field NMR spectroscopy; (iv) stable pore network and efficient fluid-transport pathways—evaluated by low-field 1D and 2D ^1^H NMR *T*_2_-*T*_2_ EXSY and *T*_1_-*T*_2_ COSY.

## 5. Conclusions

In this study, five types of nanofiber films, produced in different ratios of constituents (chitosan, marine fish collagen, fish gelatin, PVA, and PEG) were analyzed using advanced characterization techniques, such as NMR, FT-IR, XRD, and SEM. Beyond the classical SEM analysis used for the characterization of fiber diameter, length, porosity, or presence of defects, to the occasionally used FT-IR spectroscopy, it was shown that an important tool for the characterization of the entire process of fabrication from raw materials, liquid solutions to finite nanofiber nonwoven material may be represented by high-filed NMR spectroscopy or by 1D or 2D NMR relaxometry, both still extremely rarely used for these types of materials. Moreover, we demonstrated how the classical SEM analysis can be enhanced with another valence, thus adding values to SEM images, based on the use of trained ANNs for prediction of the degree of orientation of produced nanofibers with direct application, for example, to tissue engineering where uniform fiber alignment is desired for an appropriate cell orientation, but randomly oriented fibers better ensure the high nanoporous density desired for bio-membranes used for filtration. This study extends to the collection of applications of artificial intelligence (artificial neural networks/machine learning algorithms) used to predict the fiber diameter from process parameters to those which combine ANN/ML with SEM images usually used for image classification or defect detection with another one, which predicts the order degree of nanofiber orientation. In this way, the use of artificial neural networks for data analysis opens up new perspectives in the design and manufacturing of nanostructured materials. Thus, by integrating these innovative methods, more efficient and customized solutions can be developed for specific applications, with a significant impact on the research and development of advanced materials. Controlling the orientation of nanofibers plays a crucial role in optimizing their properties for specific applications, influencing key aspects such as mechanical strength, conductivity, and diffusion behavior.

## Figures and Tables

**Table 1:** Nomenclature of solutions used for the production of bio-nanofiber films by electrospinning.

Nomenclature of Dissolved Polymer Fractions Used for Electrospinning and Film Production	Dissolved Polymer Fraction 1 (Solution 1)		Dissolved Polymer Fraction 1 (Solution 2)		Fraction 1/Fraction 2
	Polymer 1 [%]	Solvent 1 [%]	Polymer 2 [%]	Solvent 2 [%]	
Ch6AA60	Chitosan 6% *w*/*v*	Acetic Acid 60%	-	-	100/0
Ch6AA90	Chitosan 6% *w*/*v*	90% Acetic Acid	-	-	100/0
Col6AA05	Collagen 6% *w*/*v*	Acetic Acid 05%	-	-	100/0
Ch6AA90(90):Col6AA05(10)	Chitosan 6% *w*/*v*	90% Acetic Acid	Collagen 6% *w*/*v*	Acetic Acid 05%	90/10
Ch6AA90(50):Col6AA05(50)	Chitosan 6% *w*/*v*	90% Acetic Acid	Collagen 6% *w*/*v*	Acetic Acid 05%	layer1/layer2
FG6H_2_O100	Fish gelatin 6% *w*/*v*	H_2_O 100%	-	-	100/0
Ch6AA90(90):FG6H_2_O(10)	Chitosan 6% *w*/*v*	90% Acetic Acid	Fish gelatin 6% *w*/*v*	H_2_O 100%	90/10
Ch6AA90(50):FG6H_2_O(50)	Chitosan 6% *w*/*v*	90% Acetic Acid	Fish gelatin 6% *w*/*v*	H_2_O 100%	50/50
PVA6AA90	PVA 6% *w*/*v*	90% Acetic Acid	-	-	100/0
Ch6AA90(90):PVA6AA90(10)	Chitosan 6% *w*/*v*	90% Acetic Acid	PVA 6% *w*/*v*	90% Acetic Acid	90/10
Ch6AA90(50):PVA6AA90(50)	Chitosan 6% *w*/*v*	90% Acetic Acid	PVA 6% *w*/*v*	90% Acetic Acid	50/50
PEG6AA90	PEG 6% *w*/*v*	90% Acetic Acid	-	-	100/0
Ch6AA90(90):PEG6AA90(10)	Chitosan 6% *w*/*v*	90% Acetic Acid	PEG 6% *w*/*v*	90% Acetic Acid	90/10
Ch6AA90(50):PEG6AA90(50)	Chitosan 6% *w*/*v*	90% Acetic Acid	PEG 6% *w*/*v*	90% Acetic Acid	50/50

The colors separates the electrospun samples based on the same dissolved polymer fraction.

## Data Availability

The original contributions presented in this study are included in the article. Further inquiries can be directed to the corresponding author.
